# ﻿Strong differentiation between amphibian communities on two adjacent mountains in the Upper Rio Pastaza watershed of Ecuador, with descriptions of two new species of terrestrial frogs

**DOI:** 10.3897/zookeys.1081.71488

**Published:** 2022-01-10

**Authors:** Juan Pablo Reyes-Puig1,2, Carolina Reyes-Puig1,3,4, Daniela Franco-Mena5,6, Lou Jost2, Mario H. Yánez-Muñoz1,2

**Affiliations:** 1 Unidad de Investigación, Instituto Nacional de Bio­diversidad (INABIO), Quito, Ecuador; 2 Fundación ECOMINGA Red de Protección de Bosques Amenazados, Fundación Oscar Efrén Reyes, Departamento de Ambiente, Baños, Ecuador; 3 Instituto de Diversidad Biológica Tropical iBIOTROP, Laboratorio de Zoología Terrestre & Museo de Zoología, Universidad San Francisco de Quito USFQ, Colegio de Ciencias Biológicas y Ambientales, Quito, Ecuador; 4 Instituto BIOSFERA Universidad San Francisco de Quito USFQ, Quito, Ecuador; 5 Ingeniería en Biodiversidad y Recursos Genéticos, Facultad de Ciencias de Medio Ambiente, Universidad Tecnológica Indoamérica, Machala y Sabanilla, Quito, EC170301, Ecuador; 6 Laboratorio de Biología Evolutiva, Colegio de Ciencias Biológicas y Ambientales COCIBA, Universidad San Francisco de Quito, Campus Cumbayá, Quito, Pichincha, Ecuador

**Keywords:** Andes, conservation, endemism, Llanganates-Sangay Ecologic Corridor, montane cloud forest, *Pristimantis*

## Abstract

We present the results of herpetological surveys in two adjacent mountains where the EcoMinga Foundation protects the cloud forest in the Upper Rio Pastaza watershed, in the Llanganates Sangay Ecological Corridor in Ecuador. A rapid assessment of the amphibian communities of the study sites reveals a diverse and heterogeneous composition, dominated by terrestrial frogs from the genus *Pristimantis*. We also identify a cryptic diversity with a significant number of candidate new species. We describe two new species of terrestrial frogs of the genus *Pristimantis*. *Pristimantismaryanneae***sp. nov.** is characterised by not having tympanum externally visible and having 2–3 subconical tubercles in the upper eyelid; and *Pristimantisburtoniorum***sp. nov.** is characterised by the presence of red colouration in hidden surfaces of the hind-limbs, tubercles on the upper eyelid, interorbital tubercle and a row of rounded tubercles along the snout to the tip and a pale red venter with dark brown mottled pattern. Our samples from the two Reserves do not share species between them, so the proportion of shared species seems to be relatively low. In addition, we highlight the importance of updating the knowledge of amphibians that are restricted to this important conservation region and comment about the threats and composition of the amphibian communities on the eastern slopes of the Upper Rio Pastaza watershed.

## ﻿Introduction

Ecological systems extending along the Cordillera Oriental of the Ecuadorian Andes harbour great amphibian diversity, with many locally-endemic species (Duellman 1978; Lynch and Duellman 1980; Yánez-Muñoz and Mueses-Cisneros 2008; Guayasamin et al. 2011; Yánez-Muñoz et al. 2013; Brito et al. 2017; Ron et al. 2020).

The ecological corridor between Llanganates and Sangay National Parks in the Upper Rio Pastaza watershed of Tungurahua and Pastaza Provinces in east-central Ecuador was declared a “Gift to the Earth” by the World Wildlife Fund in 2002, due to the great biodiversity and endemism that it houses in a small geographical area (Freile et al. 2005; Reyes-Puig and Yánez-Muñoz 2012; Ríos-Alvear and Reyes Puig 2015). Nevertheless, research conducted up to that date in the region was restricted to sites near roads and occasional collections, potentially underestimating the true biological richness of the region (Freile et al. 2005). To protect this diversity, the EcoMinga Foundation was established in January 2006 by ministerial agreement. Its main goals were to create a network of protected areas in hotspots of Andean endemism. Over the course of a decade, the EcoMinga Foundation Reserves have grown to cover about 8,000 hectares, with ten Reserves in the corridor between Llanganates and Sangay National Parks (Reyes-Puig et al. 2019) and two Reserves in western Ecuador. Nevertheless, there is no updated information on the composition and conservation status of amphibian and reptile populations in its recently-formed new reserves in the Upper Rio Pastaza watershed. Since 2008, the Herpetology Division of the Ecuadorian Museum of Natural Sciences (now the Instituto Nacional de Biodiversidad, INABIO) has carried out herpetological inventories in three Reserves of the EcoMinga Foundation in the Upper Rio Pastaza watershed: Rio Zuñag Reserve (Yánez-Muñoz et al. 2013), Río Anzu Reserve (Reyes-Puig et al. 2013) and Cerro Candelaria Reserve (Reyes-Puig et al. 2013). As a result of the material collected during these expeditions, 12 new species of amphibians have been described: *Pristimantisloujosti* (Yánez-Muñoz et al. 2010), *P.tungurahua* (Reyes-Puig et al. 2010), *Osornophrynesimpsoni* (Páez-Moscoso et al. 2011), *Pristimantisbellae* (Reyes-Puig and Yánez-Muñoz 2012), *P.ardyae* (Reyes-Puig et al. 2013), *P.marcoreyesi* (Reyes-Puig et al. 2014), *P.punzan* (Reyes-Puig et al. 2014), *P.puruscafeum* (Reyes-Puig et al. 2014), *P.pinchaque* (Reyes-Puig et al. 2014), *P.sacharuna* (Reyes-Puig et al. 2015), *Pristimantismalli* (Reyes-Puig et al. 2019) and *Noblellanaturetrekii* (Reyes-Puig et al. 2019).

The Llanganates National Park and its surroundings remain one of the least explored regions nationwide (Navarrete et al. 2016) and several of the newest EcoMinga Reserves are adjacent to its southern limits, including the Machay Reserve and Naturetrek-Vizcaya Reserve. We surveyed these two Reserves during February and March 2018 in order to determine the composition of their amphibian communities and to obtain additional material for the description of some previously-identified new species. The preliminary results are very encouraging, demonstrating the existence of several candidate new species of amphibians. These findings highlight the importance of conserving these ecosystems and continuing research in areas that have high endemism and diversity (Reyes-Puig et al. 2014; Reyes-Puig et al. 2019) and they show that, even after ten years of intensive sampling in this Andean region, our knowledge of its herpetofauna is still incomplete. In this context, we examine the amphibian diversity of the two new Reserves, we present an updated list of anurofauna from all the EcoMinga Reserves within the Llanganates-Sangay Ecological Corridor and we describe two new species of terrestrial frogs from this region.

## ﻿Materials and methods

### ﻿Study area

**Figure 1.**
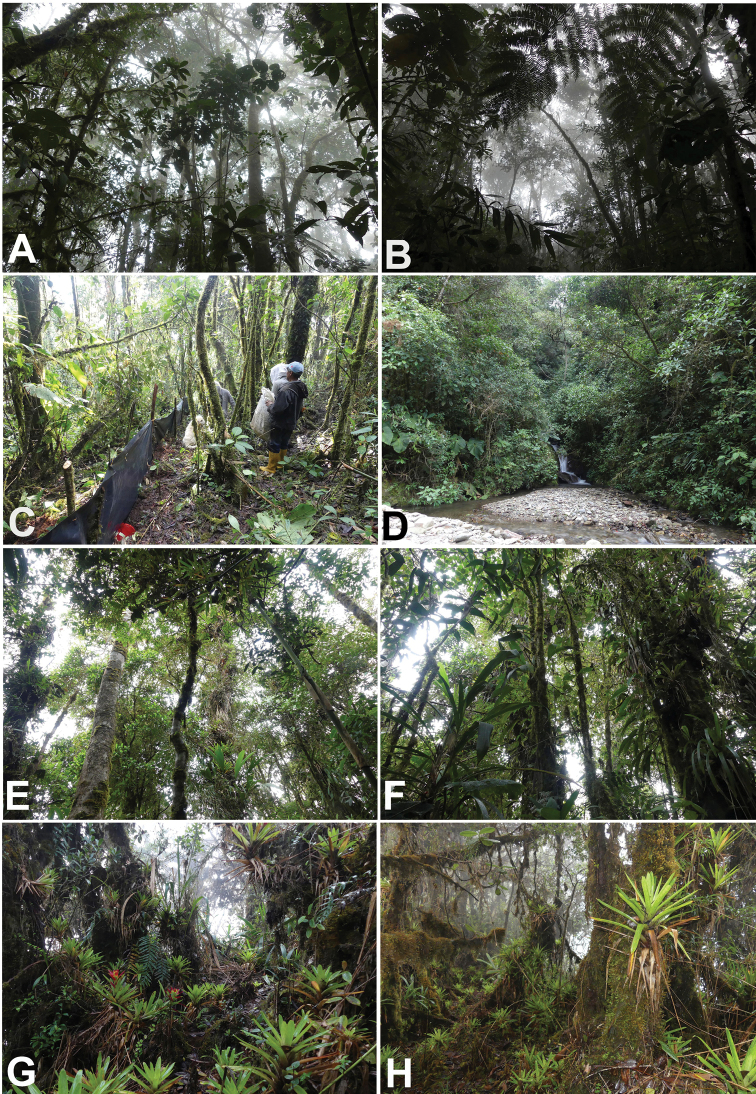
Ecosystems of the Machay Reserve and the Naturetrek-Vizcaya Reserve **A, B** point NVR 2 **C, D** point NVR 3 **E, F** point REM 1 **G, H** point REM 3. Photographs by Juan Pablo Reyes-Puig.

**Figure 2.**
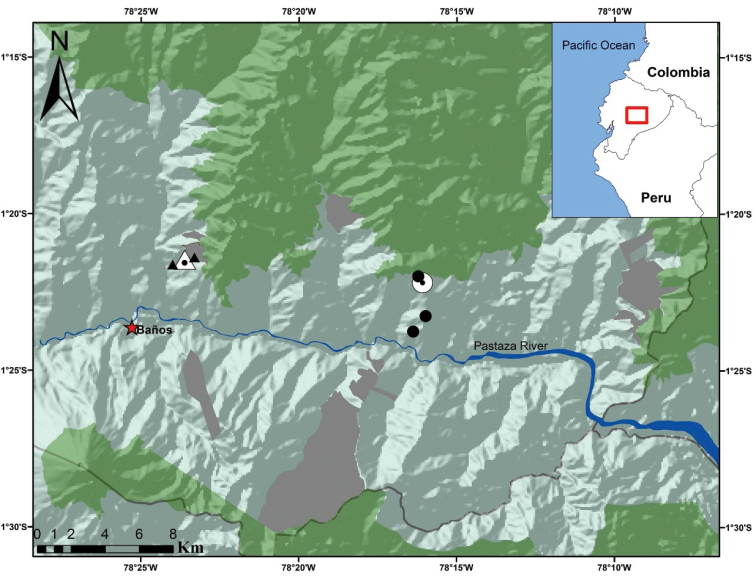
Sample points in the Naturetrek-Vizcaya Reserve (black triangles) and the Machay Reserve (black circles). The white triangle is the type locality of *Pristimantismaryanneae* sp. nov. and the white circle is the type locality of *Pristimantisburtoniorum* sp. nov. The grey shaded areas are the Reserves of the EcoMinga Foundation within the Llanganates-Sangay Corridor and the green shaded areas represent Llanganates National Park (north) and Sangay National Park (south).

We sampled the Machay Reserve and Naturetrek-Vizcaya Reserve (Table 1, Figs 1, 2), which are located in the eastern Andean slopes of Tungurahua Province, Ecuador, within the Llanganates-Sangay Ecological Corridor. Machay Reserve (MR) is located in Rio Verde Parish, Baños township, Tungurahua Province, Ecuador. The Reserve includes the valley of the Machay River and the southern slopes of Cerro Mayordomo, bordering with Llanganates National Park between 1400 and 3200 m above sea level. The sampling was carried out in the southeast slope ranging between 2200 and 3080 m above sea level, bordering the Llanganates National Park, in Baños township, Tungurahua, Ecuador. This Reserve was first formed in 2013 with the support of the World Land Trust and now contains approximately 1500 hectares of montane cloud forest. The sample points evaluated during our expeditions are described in Table 1, Fig. 1.

**Table 1.** Amphibian sampling and description of sites: Machay Reserve and Naturetrek-Vizcaya Reserve, Tungurahua Province.

Reserve	Point	Coordinates Elevation (m)	Description	Collection sources	Sampling units	Sampling effort	Days sampled	Hours per person
Naturetre-Vizcaya Reserve (NR)	NVR1	-1.358593, -78.394711; 2300 m elev.	Montane cloud forest along a mountain ridge; trees between 15 to 20 m tall with a small number of epiphytes; soil with much leaf litter, dense undergrowth and rocky outcrops.	Transects: 1000 X 2 m / 5 hours*3 persons	1	2000 m / 15 hours/person	3	15
				Pitfall 75 m	7	75 m	7	-
Naturetre-Vizcaya Reserve (NR)	NVR2	-1.356007, -78.388436; 2730 m elev.	Evergreen montane forest along a mountain ridge, trees from 20 to 25 m tall, of “Cedar” (*Cedrella*), Myrtle (*Myrcianthes*), Chigmay (*Ilex* sp.), Tarqui (Hedyosmum) and wax palm (Ceroxylum). Undergrowth dominated by Poaceae, abundant bryophytes and epiphytes.	Transects: 1000 X 2 m / 5 hours*3 persons	1	2000 m / 15 hours/person	3	15
				Pitfall 75 m	7	75 m	7	-
Naturetre-Vizcaya Reserve (NR)	NVR3	-1.360379, -78.399926; 2180 m	Riparian montane cloud forest with trees 20 m tall, such as I, *Cedrella*, *Zapotecaaculeate*, several palms and Ishnosiphon shrubs	Transects: 500 X 2 m / 5 hours*3 persons	1	1000 m / 15 hours/person	3	15
Machay Reserve (MR)	MER1	-1.395952, -78.272943; 2200 m elev.	Primary mountain mist forest on a mountain ridge with trees of 20 to 25 m tall, abundant bryophytes and epiphytes covering the trees, mainly *Magnolia* (Magnoliaceae), Myrtle (Myrcianthes), Lauraceae and different palms.	Transects: 1000 X 2 m / 5 hours*3 persons	1	2000 m / 15 hours/person	3	15
Machay Reserve (MR)	MER2	-1.387766, -78.266388; 2450 m elev.	Evergreen montane forest, with abundant epiphytes. The forest is relatively low between 15 to 20 m tall, including Pumamaqui (*Oreopanax* sp.), *Clusea*, and others. The undergrowth is dominated by Espadaña (*Neurolepis* sp.).	Transects: 1000 X 2 m / 5 hours*3 persons	1	2000 m / 15 hours/person	3	15
Machay Reserve (MR)	MER3	-1.366561, -78.270336; 3030 m elev.	Tall montane evergreen forest, with trees ranging from 15 to 20 m tall; the soil and trees are completely covered by a thick layer of bryophytes and epiphytes, especially a large number of bromeliads, *Clusea* and *Podocarpus* trees.	Transects: 1000 X 2 m / 5 hours*3 persons	1	2000 m / 15 hours/person	3	15

The Naturetrek-Vizcaya Reserve (NVR) is located within the Ulba Parish, bordering the Llanganates National Park. This Reserve was initiated in 2012 with support from the Naturetrek via the World Land Trust and new forest blocks have been added during the last few years, forming approximately 150 hectares of montane cloud forest between 2200 and 3000 m above sea level. The sample points, evaluated during our expeditions, are presented in Table 1, Fig. 1.

These two main sites are compared to four other EcoMinga Reserves in the Llanganates-Sangay Ecological Corridor: the Rio Anzu Reserve, Rio Zuñag Reserve, w – Cerro Candelaria Reserves and Chamana Reserve (Figs 1, 2).

### ﻿Fieldwork

During February and March 2018, we carried out herpetological surveys in six sampling areas (Table 1), three in the Naturetrek-Vizcaya Reserve and three in the Machay Reserve. We established two sampling transects, with lengths between 500 and 1000 m, in each area, the length of each transect depending on the topography and accessibility. The sampled altitudinal range in the Machay Reserve was between 2238 m and 3000 m (6000 m total transect length) and in the Naturetrek-Vizcaya Reserve was between 2100 m and 2900 m (5000 m total transect length). Additionally, we used pitfall traps in the Naturetrek-Vizcaya Reserve to complement the inventory.

The standardised techniques used for sampling amphibians, with their respective sampling efforts, are summarised in Table 1 and included:

**Visual Encounter Surveys (VES)** (Heyer et al.1994 and Lips et al. 2001) consist of walking through a determined area for a certain period, systematically searching for animals. We walked each sample transect with three researchers for a period of five hours nightly (between 19:00 and 24:00 hours) during six days of sampling. The order of sampling of each site, the route and the order of the observers were selected randomly, to reduce the biases that usually occur due to climatic variations from one day to another and possible biases due to the experience of the observers.

**Transects of Auditory Bands (TBA)** (Rueda et al. 2006): this technique is based on the vocalisations emitted by adult males, which are specific to each species. This method consists of counting males singing along a transect with a predetermined length (i.e. 1000 m), whose width varies according to the detection distance of the song of the focal species; that is, the maximum distance at which the animal can be heard by the observer. Individuals encountered were observed and captured and vocalising males were recorded using previously established protocols. In the case of vocalising males, the number of calls per species was recorded along intervals of the sampling unit in their respective sample area.

**Pitfall traps** (Voss et al. 1996; Rueda et al. 2016): due to the topography and accessibility of the terrain, we established in the NVR three linear transects of 75 m with 10 buckets of 20 l capacity, once every 7 m, to sample terrestrial leaf litter frogs. Buckets were checked every morning for twenty days.

**Photographic records.** We used EcoMinga reserve rangers photographs of individuals found in opportunistic sampling taken from MR. After a detailed revision of images, we registered individuals whose taxonomic identity could be confirmed from the photos.

### ﻿Specimen management

Each specimen captured was assigned a unique record number and the specimen was taken to the base camp in an individual plastic bag to confirm its sex or relative age. We recorded time of capture, type of vegetation in which it was captured, substrate, activity, and climatic conditions. To facilitate the identification of dorsal, ventral and flank patterns, each specimen was photographed with a unique code number and stored in a catalogue of photographic references.

To physically and permanently document the identification of the specimens, we deposited series of voucher specimens (Foster 2002) at the División de Herpetología (DHMECN) of the Instituto Nacional de Biodiversidad (INABIO), located in Quito, Ecuador. The specimens were fixed in formalin at 10% concentration and definitively preserved in 70% alcohol (McDairmid 1994). We identified the collected specimens in the laboratory by comparison with the reference collection at the División de Herpetología of the INABIO, specialised literature and digital wildlife repositories (Yánez-Muñoz et al. 2013, Ron et al. 2019). The identification of vocalisations was based on the acoustic base available in Bioweb Ecuador (Ron et al. 2019). The scientific nomenclature, taxonomic classification and categories for threat of extinction risk follow the proposal of Bioweb Ecuador (Ron et al. 2019).

### ﻿Morphological data and species descriptions

The description of the species follows Lynch and Duellman (1997) standard and the diagnostic characters follow the definitions and illustrations proposed by Duellman and Lehr (2009). We used the proposals of Heinicke et al. (2018) for the systematic classification of the family and we follow the putative groups of species proposed by Hedges et al. (2008) and Padial et al. (2014). Sex and age of the specimens were determined by identification of secondary sexual characteristics (nuptial pads, males with vocal slits and body size) and direct gonad inspection through dorsolateral incisions. The morphometric measures were taken with an electronic caliper (precision ± 0.01 mm, rounded to 0.1 mm), following the comments of Duellman and Lehr (2009): snout-vent length (SVL), tibia length (TL), foot length (FL), head width (HW), head length (HL), interorbital distance (IOD), width of the upper eyelid (EW), internarial distance (IND), eye-nostril distance (EN), tympanum diameter (TD) and eye diameter (ED). We recorded the colouration in life through field notes and in-field colour high-resolution images. We determined the localities, coordinates and elevations with field notes of collectors and a GPS receiver.

### ﻿Analysis of data

We generated descriptive statistics from a general double-entry diversity matrix; we tabulated the data produced in two sampling sites and then abundance-diversity figures were applied for each of the sites analysed. The data were initially tabulated and then the analyses available in the BioDiversityPro ver.2 package were applied (McAleece et al. 1997) and R (Core Team 2019). Comparisons and beta diversity analyses were based on information available for other protected areas in the zone where similar standarised sampling efforts and survey techniques were used by our team: Cerro Candelaria Reserve (Reyes-Puig et al. 2013), Río Zuñag Reserve (Yánez-Muñoz et al. 2013), Río Anzu Reserve (Reyes Puig et al. 2013) and Chamana Reserve (in preparation). The degree of similarity between sampled sites was calculated using a cluster analysis of similarity, based on the Jaccard Coefficient.

The standardised samples, obtained from the transects, were further analysed to compare the diversities of the Machay and Naturetrek-Vizcaya Reserves. Two types of rarefaction were applied, to correct for between-reserve differences in number of individuals, sampling effort and sample completeness (Chao and Jost 2012; Chao et al 2016).

An exploratory post-hoc linear regression of log_10_SVL versus elevation was conducted on the eighteen adult females from the genus *Pristimantis* (the more abundant genus) of both sites, regardless of species. This analysis was not intended to test a previously-established hypothesis, since it was carried out later; however, we wanted to inspect the general pattern and describe it for future studies related to the variation in body size.

The species recorded were classified according to distribution, threat level and reproductive strategies using the following categories:

**Distribution Range: IN** = Introduced, **NE** = Not Evaluated, **WN** = Wide Neotropical distribution, **EC** = Endemic of Ecuador, **AN** = Endemic from the Andes.

**Extinction Risk** IUCN (2019): **NE** = Not Evaluated, **LC** = Least Concern, **NT** = Near Threatened, **VU** = Vulnerable, **EN** = Endangered and **CR** = Critically Endangered.

***Reproductive strategies***: We use the classification system of reproductive modes or strategies of Duellman and Trueb (1994) and Haddad and Prado (2005).

## ﻿Results

**Figure 3.**
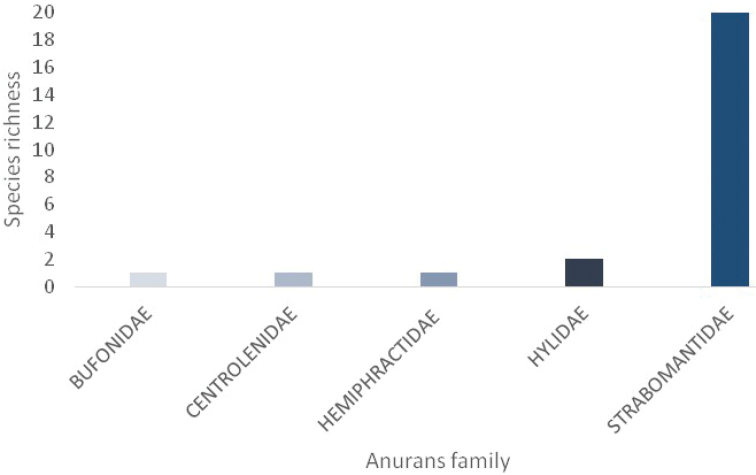
Taxonomic composition of the anurofauna: Naturetrek-Vizcaya and Machay Reserves.

During surveys, we recorded 97 individuals of anurans grouped into seven genera and five families (Fig. 3); 94 captured and three by auditory records; to see absolute abundance, please see Appendix 1. The frogs recorded were found along an altitudinal gradient from 1990 m to 3020 m in elevation. Terrestrial frogs (Strabomantidae) made up 91.3% of the taxonomic composition; the remaining families include true toads (Bufonidae), marsupial frogs (Hemiphractidae), tree frogs (Hylidae) and glass frogs (Centrolenidae) which each contributed one or two species to the total composition (Fig. 3).

**Figure 4.**
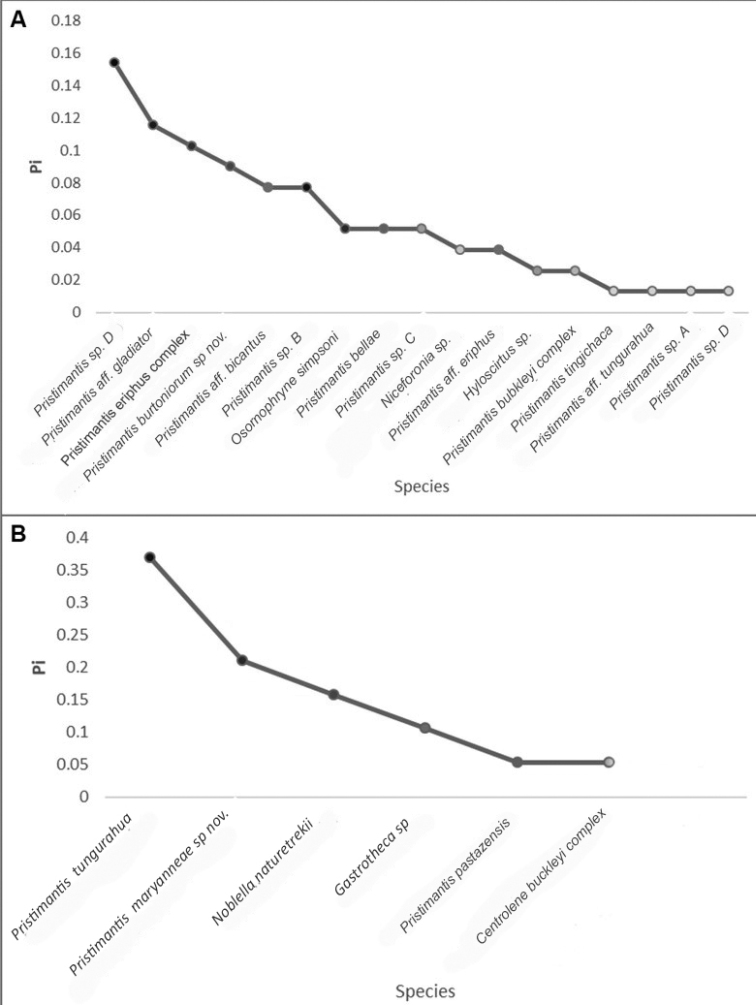
Curve of dominance and diversity of the amphibians from: **A** Machay Reserve and **B** Naturetrek-Vizcaya Reserve.

In the Machay Reserve, 78 individuals were registered; the community was composed of 17 species, led by terrestrial frogs (Fig. 4). The dominant species was the rain frog *Pristimantis* sp. D, followed by Pristimantisaff.gladiator, *P.eriphus complex*, *Pristimantisburtoniorum* sp nov, P.aff.bicantus and *Pristimantis* sp. B. The remaining 38.4% of the amphibian composition was represented by 12 species with abundance between one and four individuals (Fig. 4).

**Figure 5.**
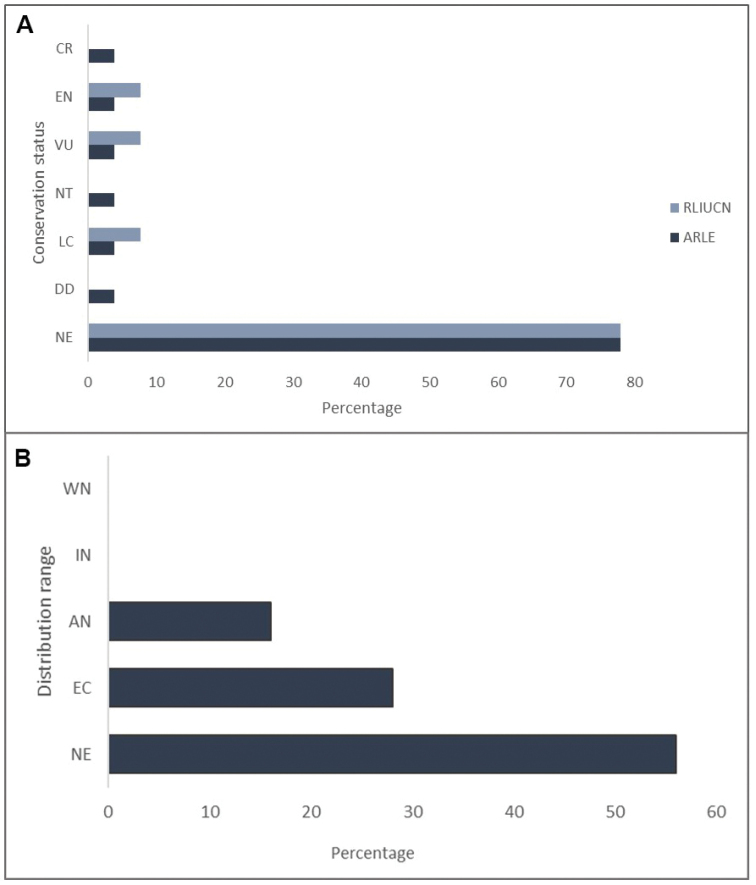
**A** Conservation status categories of the amphibian composition from the Machay Reserve and the Naturetrek-Vizcaya Reserve, under the IUCN Red List and ARLE Amphibian Red List Ecuador (IUCN 2012; Ron et al. 2019) **B** Distribution ranges of amphibians from MR and NVR: IN = Introduced, NE = Not Evaluated, WN = Wide Neotropical distribution, EC = Endemic to Ecuador, AN = Endemic to the Andes.

In the Natutretrek Viscaya Reserve, 19 individuals were registered, including captured and calling individuals; the community was composed of six species. The number of individuals registered comprise 22% of the total number of individuals recorded for the two Reserves. The dominant species was the rain frog *Pristimantistungurahua*, followed by *Pristimantismaryanneae* and *Noblellanaturetrekii*; the remaining species had abundances between one and two individuals (Fig. 5).

### Patterns of distribution and conservation status

80% of the recorded species do not have a regional or global conservation status. Many of these taxa are not formally described; thus, categorising their range distribution is work that must be done in the future. The remaining 20% of the species correspond to Vulnerable, Endangered and Critically Endangered species. Nonetheless, some taxa are species complexes and need their taxonomical relationships clarified. On the other hand, all of the species with a taxonomic identity (i.e. formally described) are restricted to the eastern Andean slopes and 55.5% of these species are completely restricted to the upper Rio Pastaza watershed within Tungurahua Province (Fig. 5). There are no recorded species with wide Neotropical distribution, nor any introduced species in the study sites (Fig. 5).

**Figure 6.**
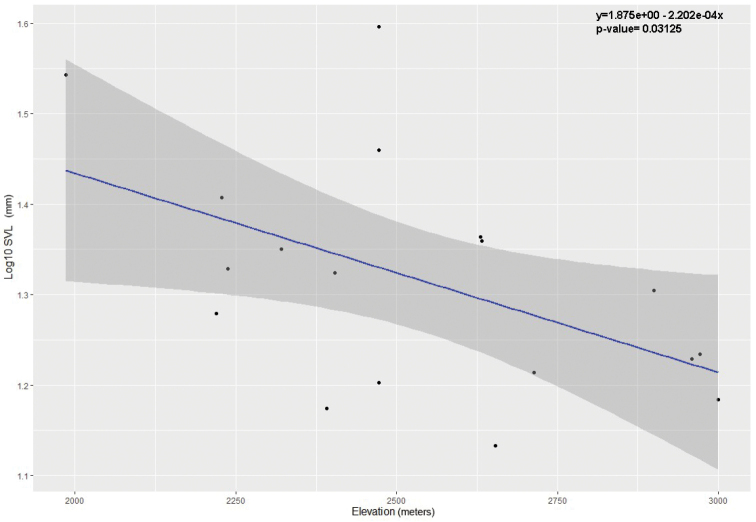
Trend between the snout-vent length (SVL) and elevation in collected adult amphibian females from the Machay Reserve and Naturetrek-Vizcaya Reserve.

We found that log_10_SVL (in mm) of individual adult females (all given equal weight, without regard to species) showed a weak negative correlation with elevation (in metres). The least squares line (with 95% confidence intervals) had a slope of -0.00022 (-0.00002 to -0.0004) and an intercept (extrapolated log_10_SVL at sea level) of 1.9 (1.36 to 2.37); r2 = 0.3 and p = 0.03. We give this p-value only for reference; since this was a post hoc analysis, p-values do not have a simple interpretation. The fitted line predicts a SVL of 27.5 mm (21 mm to 36 mm) at 2000 m elevation and a SVL of 17 mm (13 mm to 21 mm) at 3000 m elevation (Fig. 6).

### Reproductive strategies

**Figure 7.**
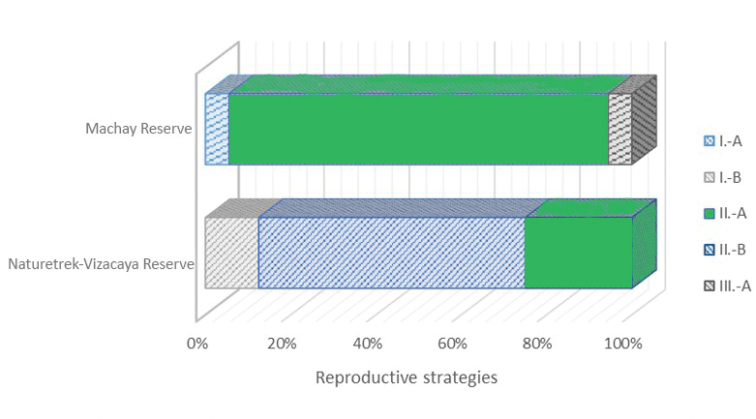
Anuran reproductive strategies within Naturetrek-Vizcaya Reserve and Machay Reserve. Types of reproductive strategies **I.-A** Eggs hatch in exotropic tadpoles that are carried to lentic water. Eggs and feeding tadpoles in lentic water **I.-B** Eggs hatch in exotropic tadpoles that are taken to lotic waters. Eggs and feeding tadpoles in lotic water **II.-A** Eggs with direct development, completely formed hatched frogs **II.-B** Eggs imbedded in dorsum of aquatic female; eggs hatch into froglets **III.-A** Ovoviviparous; nutrition is provided by the yolk.

Five breeding modes (Haddad and Prado 2005) were determined for amphibians on the Machay Reserve and Naturetrek-Vizcaya Reserve (Fig. 7). A total of 80.7% of the reported species have direct development reproductive mode (Fig. 7). The remaining percentage corresponds to growth and development of tadpoles associated with water bodies (Fig. 7).

#### ﻿Estimation of species richness

Our standardised transect sampling on the two mountains revealed both a higher density of individuals and higher number of species in the Machay Reserve. In the Machay Reserve, there was a density of 1.7 individuals per sampling hour per person, while in the Naturetrek-Vizcaya Reserve, the density was only about 1/4 of this, at 0.4 individuals per sampling hour per person. Twelve species were observed on transects in the Machay Reserve, while only four species were observed on transects in the Naturetrek-Vizcaya Reserve. The transect sample from the Machay Reserve has a completeness or sample coverage of 87%, while that of the Naturetrek-Vizcaya Reserve has a completeness or sample coverage of 83%. Sample coverage is the proportion of the community’s population that belongs to species detected by the sample (Chao and Jost 2012).

To determine whether the higher diversity of the Machay Reserve was real or was merely due to the higher density of individuals encountered there, we examined the rarefaction curves for the two sites. The standard rarefaction or species accumulation curve for the Machay sample was always significantly above the curve for the Naturetrek-Vizcaya sample (Fig. 8a). The same was true for the curve of species accumulation versus sample completeness (Fig. 8b), which is a measure based on the slope of the standard species accumulation curve (Chao and Jost 2012). The diversity discrepancy between the two sites, therefore, appears to be real and not just due to the smaller number of individuals found on the Naturetrek-Viscaya Reserve.

**Figure 8a.**
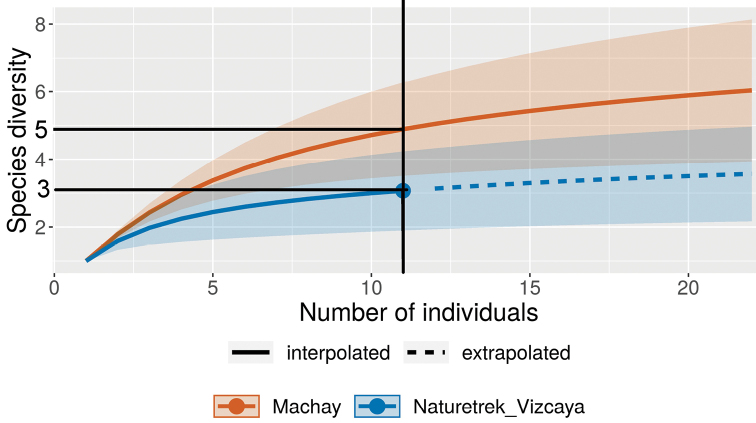
Interpolated and extrapolated species accumulation curves versus number of individuals for the Machay and Naturetrek-Vizcaya Reserves. The black vertical line marks the number of individuals at which the richness of each are compared on each curve (this is the size of the smallest sample). At this sample size (n =11), the sample from Naturetrek-Viscaya would on the average have three species, while a sample of the same size from Machay would be expected to have five species.

**Figure 8b.**
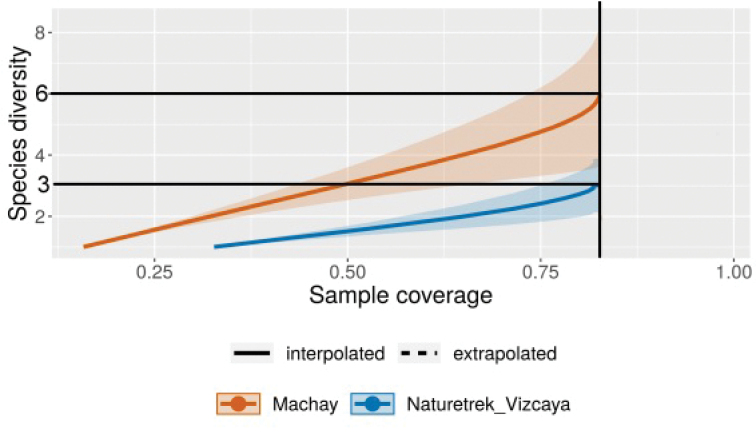
Interpolated and extrapolated species accumulation curves versus sample coverage (percent of the population represented by species in the sample) for the Machay and Naturetrek-Vizcaya Reserves. The black vertical line marks the number of individuals at which the richness of each are compared on each curve (this is the coverage of the least complete sample). At this coverage, the sample from Naturetrek-Viscaya would on the average have three species, while a sample of the same coverage from Machay would be expected to have six species.

### Beta diversity

**Figure 9.**
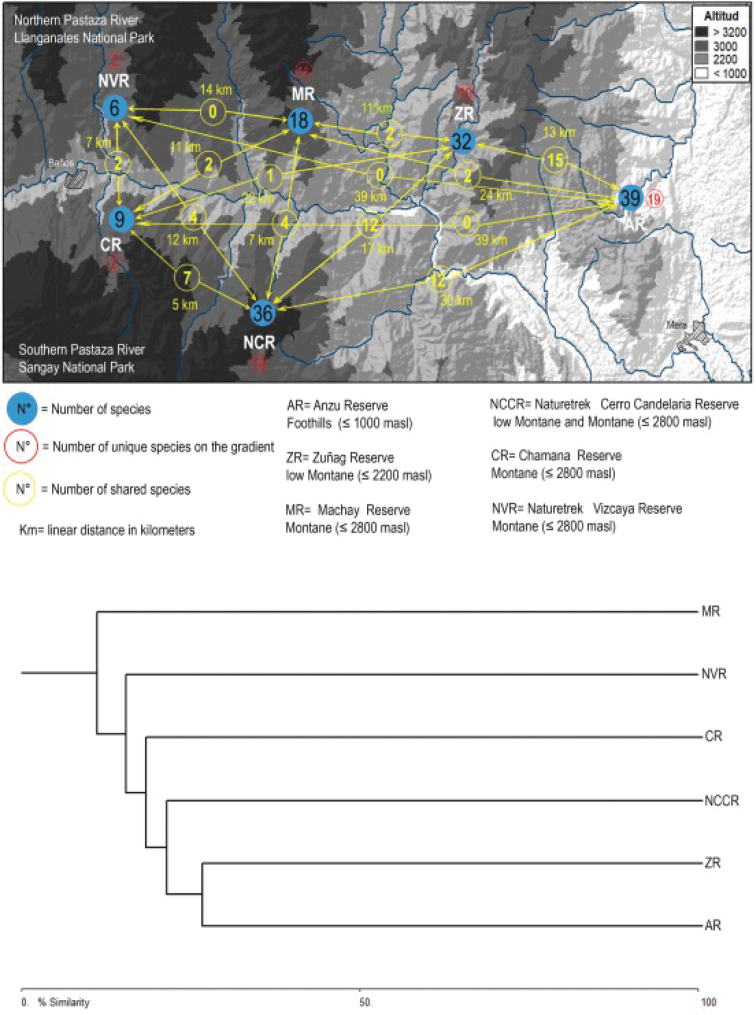
Beta diversity of the anuran communities in the Upper Rio Pastaza watershed. Above: Similarity diagram. Below: Cluster analysis based on the Jaccard analysis for the six studied localities in the Upper Rio Pastaza watershed.

The amphibian community at the six studied localities of the upper Rio Pastaza watershed was heterogeneous (Appendix 2, Fig. 9). The locality at the lowest elevation, the Anzu Reserve which ranges from 1150 m – 1250 m, is the most diverse. Areas with large elevation gradients (Naturetrek-Cerro Candelaria Reserves and Zuñag Reserve), in transition zones between the headwaters and the mid-basin of the Pastaza River (1400 m to 3800 m), also stood out for their high number of species (Table 2, Fig. 9).

**Table 2.** Comparison matrix of diversity data in six EcoMinga Reserves of the Upper Rio Pastaza watershed. In bold and white, number of species per site. Above the species line, in italics, percentage of shared species. Below the species line, linear distance in kilometres between sites compared.

Location	Sites	AR	ZR	MR	NVR	CR	NCCR	Unique species per site
**Northern Pastaza**	**AR**	**39**	*11*	*4*	*0*	*0*	*19*	19
**Northern Pastaza**	**ZR**	13	**32**	*11*	*0*	*3*	*21*	10
**Northern Pastaza**	**MR**	24	11	**18**	*0*	*8*	*4*	11
**Northern Pastaza**	**NVR**	39	25	14	**6**	*15*	*4*	2
**Southern Pastaza**	**CR**	30	17	7	12	**9**	7	2
**Southern Pastaza**	**NCCR**	35	22	11	7	5	**36**	10
**Lower elevation limit in metres**		1050	1400	1500	2300	2300	1500	–
**Upper elevation limit in metres**		1300	2200	3030	2700	2700	3860	–
**Coordinates**		-1.358593°, -78.394711°	-1.358593°, -78.394711°	-1.366561°, -78.270336°	-1.358593°, -78.394711°	-1.358593°, -78.394711°	-1.358593°, -78.394711°	

**Figure 10.**
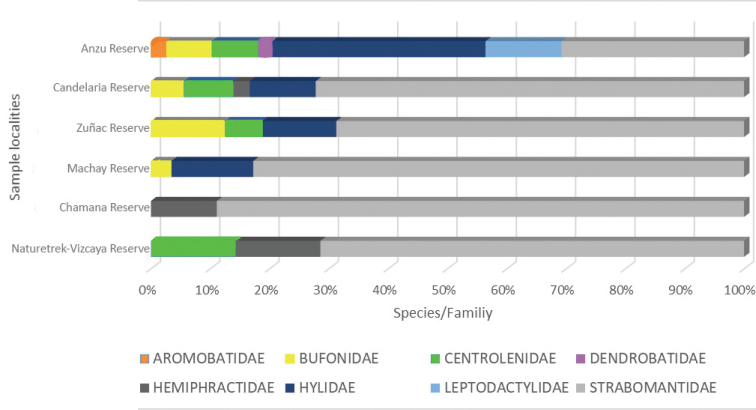
Species by amphibian family in six EcoMinga Reserves of the Upper Rio Pastaza watershed.

The number of species and number of individuals per family in each locality were highest in the Anzu Reserve and Naturetrek-Cerro Candelaria Reserves. Rain frogs (Strabomantidae) are the predominant family in all six Reserves. At a regional scale, tree frogs (Hylidae) have significantly reduced diversity, compared to the Terrarana (Strabomantidae) and the proportions of the remaining families, such as Bufonidae, Centrolenidae, Leptodactylidae, Hemiphractidae, and Dendrobatidae, are not constant in the Reserves of the Upper Rio Pastaza watershed (Fig. 10).

The degree of similarity according to the Jaccard Index reflects that the composition amongst the six localities only shared 11% similarity, Zuñag and Anzu Reserves are more similar, sharing only 27% of their species and present high diversity of elements typical from lowland Amazonian tropical forest mixed with montane species. The cluster analysis defines highly heterogeneous communities between Reserves, showing different composition of anuran assemblages delimited by topography north and south to the Pastaza River and its proximity to the highland Andes or the Amazon Basin (Figs 9, 10).

## ﻿Species accounts

### ﻿BUFONIDAE

#### ﻿ Osornophryne simpsoni

Taxon classificationAnimaliaAnuraBufonidae

Páez-Moscoso, Guayasamin & Yánez-Muñoz, 2011

C89A4C39-E49F-59C7-A5C3-870E4A48A768

Figure 11

##### Remarks.

We recorded three adult female specimens (DHMECN 14412-14413-14414) with body sizes between 26.58 mm and 33.57 mm collected at night on bromeliad leaves, palms and Neurolepis (Poaceae), between 40 cm and 170 cm from the ground and a juvenile (DHMECN 14415) with body size 12.38 mm, collected in a bromeliad 20 cm from the ground. According to the original species description, *O.simpsoni* was known only from two localities in the upper Rio Pastaza watershed and Cordillera Abitahua (Páez et al. 2012). The record in Machay Reserve corresponds to the third locality of the species within Ecuador. This taxon is characterised by having Toes IV and V longer than Toes I–III, a short and rounded snout with a small rostral papilla and conical pustules on flanks. This species of plump toad inhabits the high montane forests of the Machay Reserve, between 2430 and 2490 m in elevation, this forest being characterised by low trees with abundant bryophytes and bromeliads. Under direct manipulation, this species tends to escape with slow movements and lets itself fall in a ball-like position.

**Figure 11.**
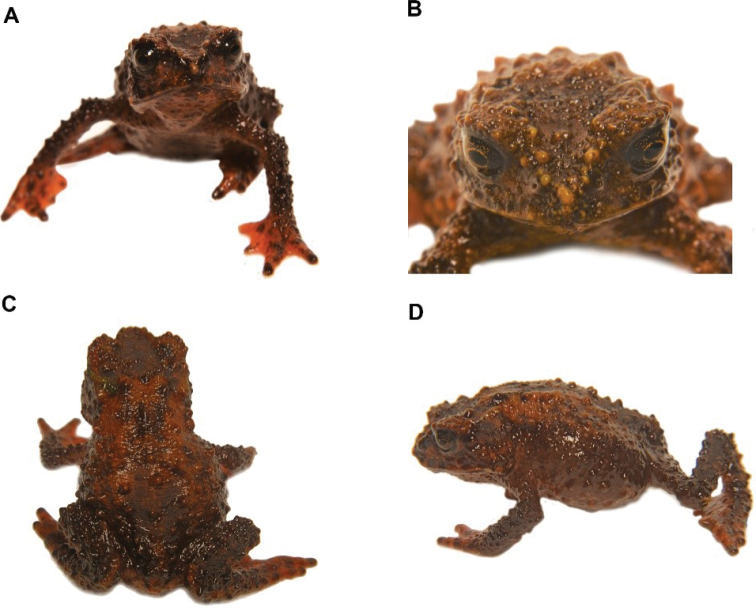
*Osornophrynesimpsoni*, male **A, B** Frontal view of the head **C** dorsal view and **D** view of the flanks. (DHMECN 14412). Photographs by Mario H. Yánez-Muñoz

### ﻿CENTROLENIDAE

#### ﻿ Centrolene buckleyi

Taxon classificationAnimaliaAnuraCentrolenidae

complex

8745346E-20CF-5B0A-9A32-26B43975ED37

Figure 12

##### Remarks.

This species was recorded in riparian vegetation through previous observations in nearby localities, by auditory and a recent voucher specimen record (DHMECN 16217) in montane forests of the Naturetrek-Vizcaya Reserve from 2270 to 3000 m elevation. According to a photograph, the specimen may be associated with the *C.buckleyi* species group (Yánez et al. 2010). Previous analyses of the group’s phylogenetic relationships show a high cryptic diversity in the species complex (Amador et al. 2018). Therefore, this could correspond to a potentially new species, though additional collections and phylogenetic analysis of these populations is necessary to determine its status. Centrolenid frogs are generally known to live on clean water streams, its presence on Naturetrek-Vizcaya reflecting good ecological conditions and no water pollution in the zone.

**Figure 12.**
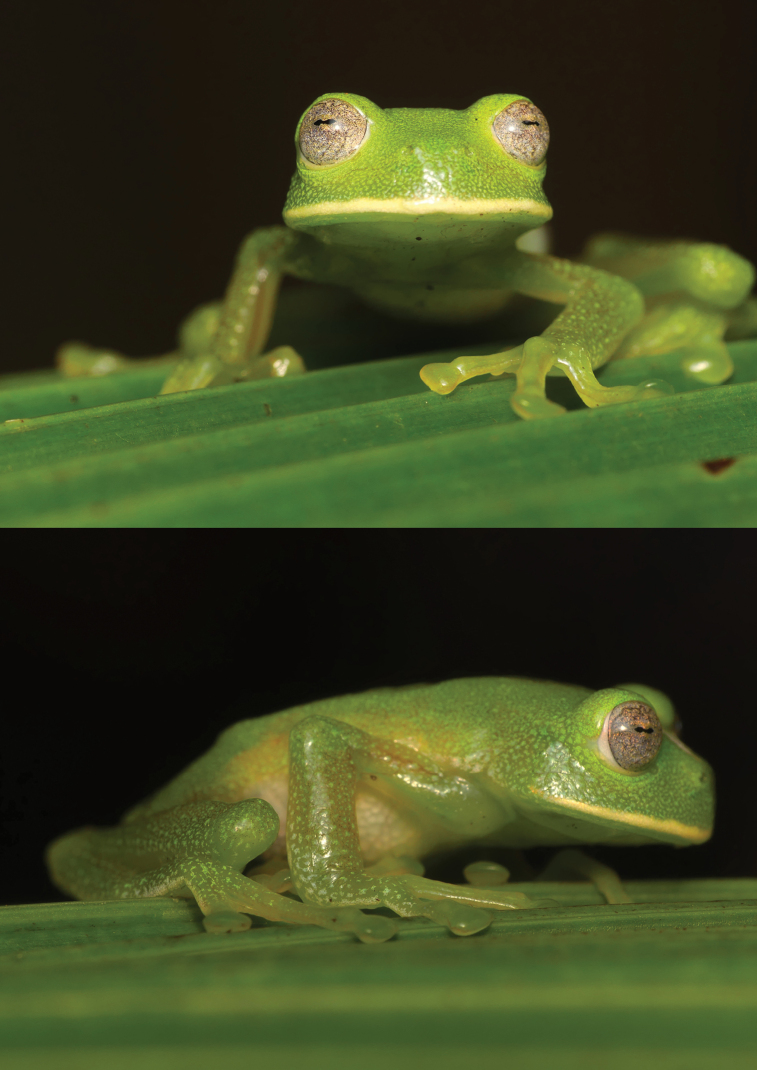
*Centrolenebuckleyi* complex, frontal and lateral views (DH-MECN 16217). Photographs Juan Pablo Reyes Puig.

### ﻿HEMIPHRACTIDAE

#### ﻿ Gastrotheca

Taxon classificationAnimaliaAnuraHemiphractidae

sp.

CBABD27E-50BF-57F5-BCB5-C47F42D5732F

##### Remarks.

The marsupial frogs of the genus *Gastrotheca* were recorded by audio records, but were difficult to capture, in the cliffs and southern montane forests of the Naturetrek-Vizcaya Reserve at 2270 m elevation. The auditory records analysed are similar to those issued by *Gastrothecariobambae* (Fowler, 1913) which has been recorded a few kilometres from Naturetrek-Vizacaya Reserve in the locality of El Triunfo (Ortiz and Morales 2000), confirming the record would broaden the distribution range of the species. *G.testudinea* (Jiménez de la Espada, 1871) has been recorded near this locality in agriculture and forest lands, so there is a possibility that both species are present in the area.

### ﻿HYLIDAE

#### ﻿ Hyloscirtus sp. larynopygion

Taxon classificationAnimaliaAnuraHylidae

species group

ED2697FA-3766-5FB6-B01A-58E0F7976562

Figure 13

##### Remarks.

We recorded a female specimen (DHMECN 14416) with a body size of 73.71 mm and a male (DHMECN 14549) with a body size of 54.28 mm, collected in bromeliad leaves between 40 and 60 cm from the ground, in montane forest of the Machay Reserve at 3020 m elevation. They correspond to a candidate new species of the genus *Hyloscirtus* in the *H.larynopigion* species group. The distinctive dark brown body with scattered bright red dorsal and ventral spots in the female (DHMECN 14416) and the irregular mustard-brown dorsal marks and black flanks in the male (DHMECN 14549), differentiate it from any other congeneric species in the eastern Andean slopes of Ecuador. The analysed material fills a gap in the distribution of the genus *Hyloscirtus*, specifically of the *H.larynopigion* species group, within the south-central area of the eastern Ecuadorian Andes. This finding represents the first record of a member of the *Hyloscirtuslarynopigion* species group in Tungurahua Province. The species presents a marked sexual dimorphism between males and females as mentioned before. A formal description of this new species is currently under preparation.

**Figure 13.**
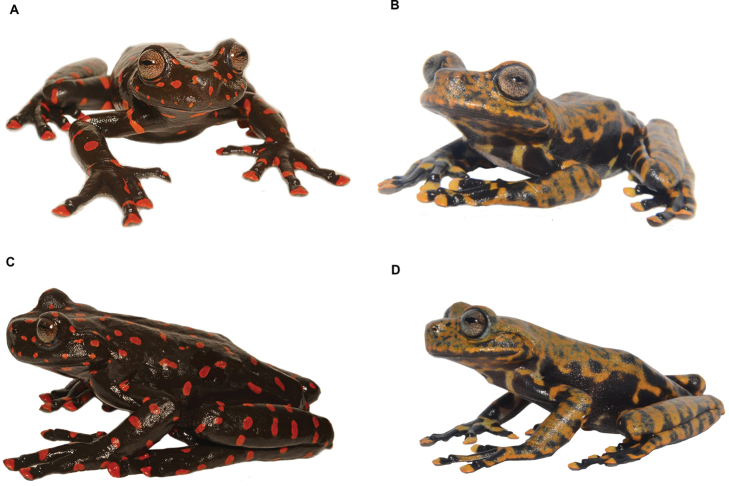
*Hyloscirtus* sp. nov. Left. Female (DHMECN 14416)-Right. Male (DHMECN 14549). **A, B** Frontal view **C, D** Lateral view. Photographs by Mario H. Yánez-Muñoz.

### ﻿STRABOMANTIDAE

#### ﻿ Niceforonia

Taxon classificationAnimaliaAnuraStrabomantidae

sp.

3389421F-DE69-5326-AB98-FD36DE2E5CDD

Figure 14

##### Remarks.

These records correspond to a candidate new species; this is a new terrestrial frog very different from the *Niceforoniaelassodisca* expected to be found in the region. Two female specimens (DHMECN 14417-14418) with body sizes of 16.36 mm to 16.95 mm, one male individual (DHMECN 14419) with body size of 19.15 mm and one immature specimen (DHMECN 14488) with body size of 12.73 mm, were collected between dry leaves of *Clusia* and bulrush in the leaf-litter in montane forest of the Machay Reserve between 2480 and 2960 m elevation. Its uniformly cream-coloured ventral pattern clearly distinguishes it from *H.elassodisca*. *Niceforoniaelassodisca* has a dark venter and no defined subocular and supratimpanic cantal bands, while *Niceforonia* sp. has a defined subocular and supratimpanic cantal band, as well as a dark brown anal ornamentation.

**Figure 14.**
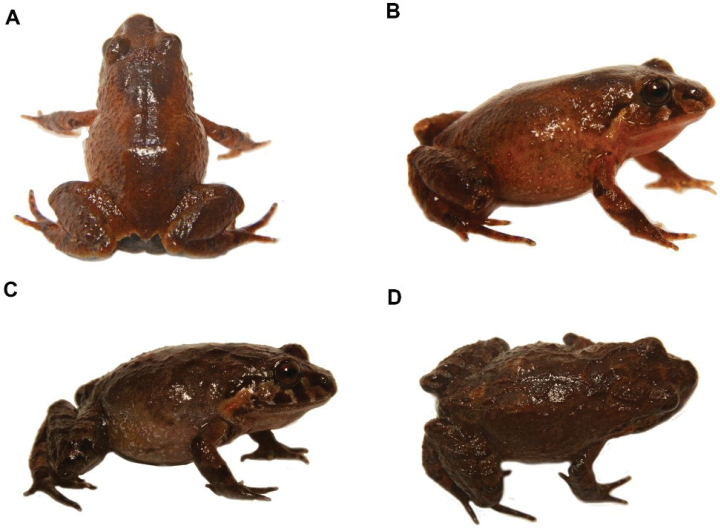
*Niceforonia* sp. Up row female (DHMECN 14418), lower row male (DHMECN 14419). **A, B** Male dorsal and lateral view **C, D** Female Dorsal and lateral view. Photographs by Mario H. Yánez-Muñoz.

#### ﻿ Noblella naturetrekii

Taxon classificationAnimaliaAnuraStrabomantidae

Reyes-Puig et al. 2019

15B8079A-8E94-56A9-82A0-EF1DD15A08AB

Figure 15

##### Remarks.

A recently described species (Reyes-Puig et al. 2019), we recorded a male specimen (DHMECN 14437) with body size of 17.44 mm and a female specimen (DHMECN 14420) with body size of 14.92 mm, collected in the Naturetrek-Vizcaya Reserve at 2390 m elevation. These specimens correspond to the first records of the genus over 2000 m above sea level in the eastern Andean slopes of Ecuador. Both specimens were captured in pitfall traps. This species differs from its congeners by the presence of a differentiated tympanic membrane and a weakly-defined tympanic annulus, eyelids with rounded tubercles, blackish-dark brown ventral colouration scattered with little white dots and the absence of papillae at the tip of the fingers and toes, unlike *Noblellacolomai*, *N.personina*, *N.myermecoides*, *N.lochites* and *N.heyeri* that have visible tympanic annuli and pale or colourful bellies.

**Figure 15.**
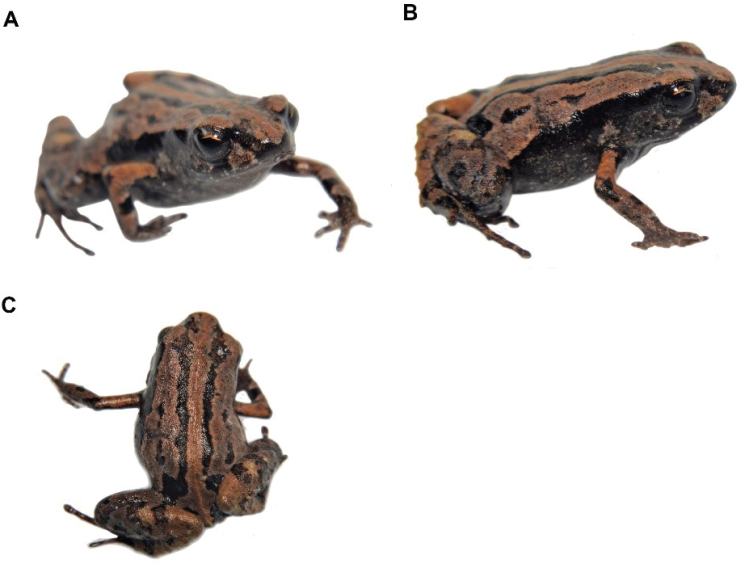
*Noblellanaturetrekii*, Male Paratype (DHMECN 14420) **A** frontal view **B** lateral view **C** dorsal view. Photographs by Mario H. Yánez-Muñoz.

#### ﻿ Pristimantis bellae

Taxon classificationAnimaliaAnuraStrabomantidae

Reyes-Puig & Yánez-Muñoz, 2012

347E9A15-3C7C-5026-A31E-6242F275E84C

Figure 16

##### Remarks.

We collected two female specimens (DHMECN 14423-14424) with body sizes of 21.33 mm up to 22.40 mm and two male specimens (DHMECN 14421-14422) with body sizes of 15.58 mm up to 16.73 mm; the specimens were found on bush, palm and cyclanth leaves between 20 cm and 50 cm from the ground in montane Machay Reserve forests at 2290 m elevation. This species is characterised by having a distinctive pattern of irregular white marks on the black venter, conical tubercles on the upper eyelid, one interorbital tubercle and a row of ulnar and tarsal tubercles.

**Figure 16.**
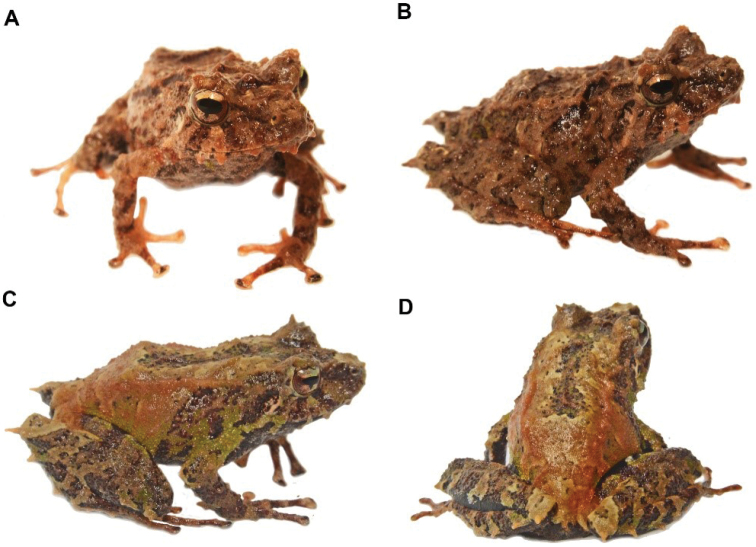
*Pristimantisbellae*,variation in females **A** frontal view **B** lateral view (DHMECN 14430) **C** lateral view and **D** dorsal view (DHMECN 14424). Photographs by Mario H. Yánez-Muñoz.

#### ﻿ Pristimantis pastazensis

Taxon classificationAnimaliaAnuraStrabomantidae

Andersson, 1945

C54DB487-3F03-53B1-AD96-FBD9A4473AD2

Figure 17

##### Remarks.

This record extends the known distribution of the species, since the available records had been restricted to a few localities near the Tungurahua Volcano (Reyes Puig et al. 2014; Ron et al. 2019). We documented a juvenile specimen (DHMECN 14425) with body size 15.05 mm that was collected on an anthurium leaf 160 cm from the ground, in mountain forests of the Naturetrek-Vizcaya Reserve at 2100 m elevation. *Pristimantispastazensis* is characterised by having a rostral papilla, snout subacuminate in dorsal view and for not having pungent tubercles on the eyelids.

**Figure 17.**
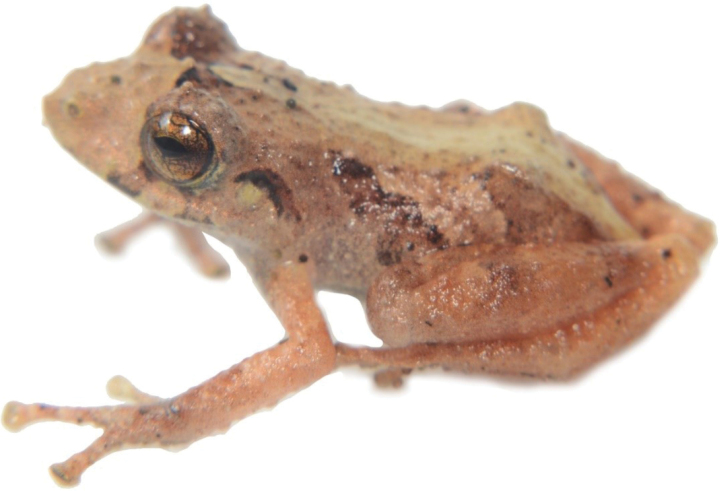
*Pristimantispastazensis*, Male (DHMECN 14425), lateral view. Photographs by Mario H. Yánez-Muñoz.

#### ﻿ Pristimantis tinguichaca

Taxon classificationAnimaliaAnuraStrabomantidae

Brito M, Ojala-Barbour, Batallas R & Almendáriz C, 2016

35D18BFB-4779-5B8B-B1B7-614F36E47104

Figure 18

##### Remarks.

We recorded a female specimen (DHMECN 14426) with body size of 28.83 mm collected on a palm leaf in the montane forests of the Machay Reserve at 2290 m elevation. This specimen corresponds to the first record of *P.tinguichaca* on the north side of the Rio Pastaza and extends its altitudinal distribution in the Province of Tungurahua from the previous record of 2470 m (Brito et al. 2016; Franco-Mena et al. 2019). This species can be distinguished by the presence of small conical tubercles on the upper eyelids and heels and by its red-coloured iris.

**Figure 18.**
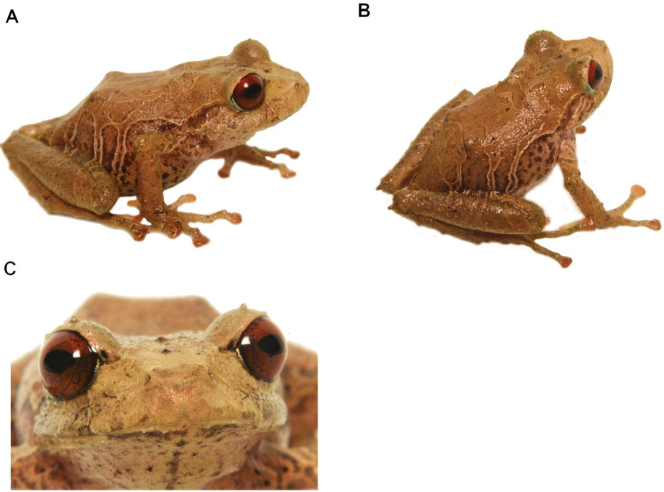
*Pristimantistinguichaca*, Female (DHMECN 14426) **A** lateral view **B** dorsal view **C** frontal view **D** dorsal view. Photographs by Mario H. Yánez-Muñoz.

#### ﻿ Pristimantis tungurahua

Taxon classificationAnimaliaAnuraStrabomantidae

Reyes-Puig, Yánez-Muñoz, Cisneros-Heredia & Ramírez, 2011

7441587E-B4D3-5BBF-9E3F-11BD58A6733E

Figure 19

##### Remarks.

We documented three female specimens (DHMECN 14427, 14428, 14429, 14433) with body sizes of 20.15 mm up to 22.87 mm, two male specimens (DHMECN 14430, 14431) with body sizes of 15.87 mm up to 16.77 mm and one juvenile specimen (DHMECN 14432) with a body size of 14.02 mm; the specimens were collected on leaves of ferns and shrubs between 30 cm and 40 cm from the ground and dry sticks between leaves at ground level; some of them were captured in pitfall traps. These specimens and others in the Chamana Reserve correspond to the first records since the original description (Reyes-Puig et al., in preparation); these records extend the limits of altitudinal distribution to the range of 2400 m to 2900 m. This species can be easily distinguished by its distinctive red salmon colouration on the groin, ventral surfaces of the limbs and by the presence of dorsolateral folds.

**Figure 19.**
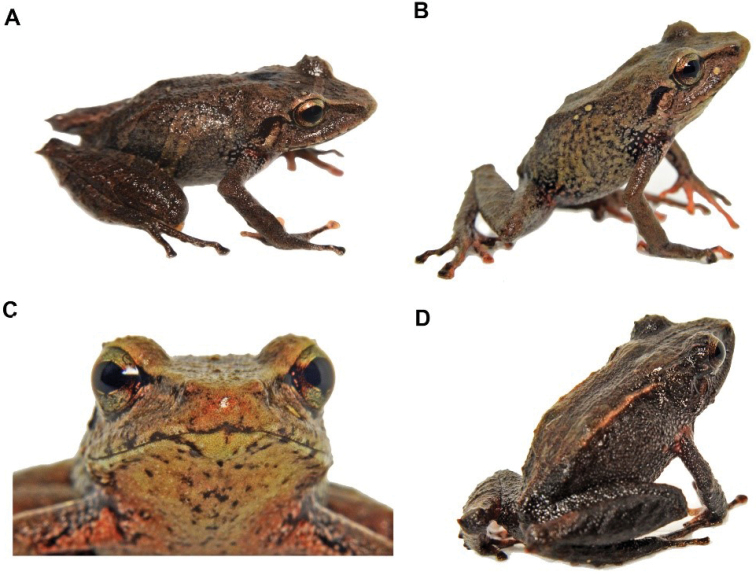
*Pristimantistungurahua*, Females **A** lateral view (DHMECN 14428) **B** lateral view **C** frontal view (DHMECN 14427) **D** dorsal view (DHMECN 14428). Photographs by Mario H. Yánez-Muñoz.

#### ﻿ Pristimantis buckleyi

Taxon classificationAnimaliaAnuraStrabomantidae

complex

8CFC95CA-0609-543D-B846-F7AB52707E92

Figure 20

##### Remarks.

Two specimens (DHMECN 14434-14435) of body sizes between 21.64 mm and 27.97 mm were collected on a *Clusia* leaf 110 cm from the ground, in the montane forests of Cerro Mayordomo at 3000 m elevation. This is species is characterised by low dorsolateral folds and an areolate venter.

**Figure 20.**
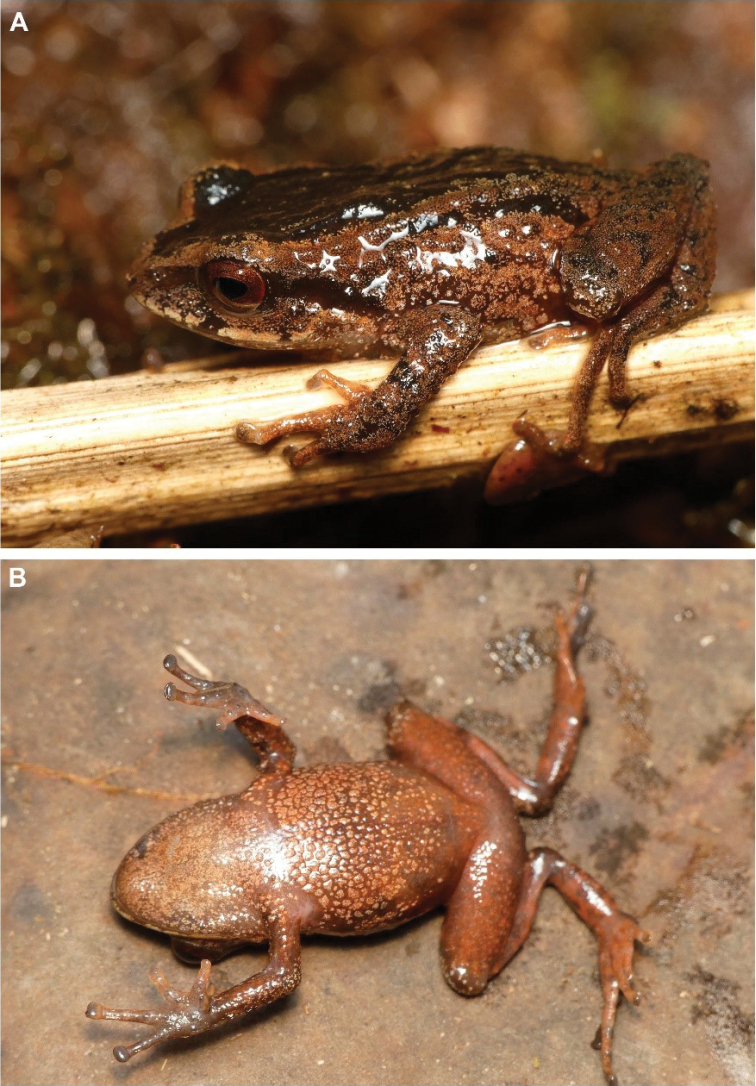
*Prisitmantisbuckleyi* complex. (DHMECN 14435) **A** lateral view **B** ventral view. Photographs by Lou Jost.

#### ﻿ Pristimantis eriphus

Taxon classificationAnimaliaAnuraStrabomantidae

complex

651F8F94-5957-552C-A118-F5A3D0AF891B

Figure 21

##### Remarks.

One female specimen (DHMECN 14436) with body size of 25.53 mm, a male specimen (DHMEN 14437) with body size of 11.37 mm and five male specimens (DHMECN 14438- 14439-14487-14440-14441) of body sizes of 15.3 mm up to 22.91 mm, were collected on bush leaves and branches 170 cm from the ground, in Machay Reserve montane forests at elevations ranging from 2230 m to 2310 m. This species had already been registered in other EcoMinga Reserves, such as the Cerro Candelaria Reserve and the Río Zuñag Reserve. This species is characterised by having a conical tubercle on the upper eyelid and the heel and by having coppery to red iris in females, dark yellow in males.

**Figure 21.**
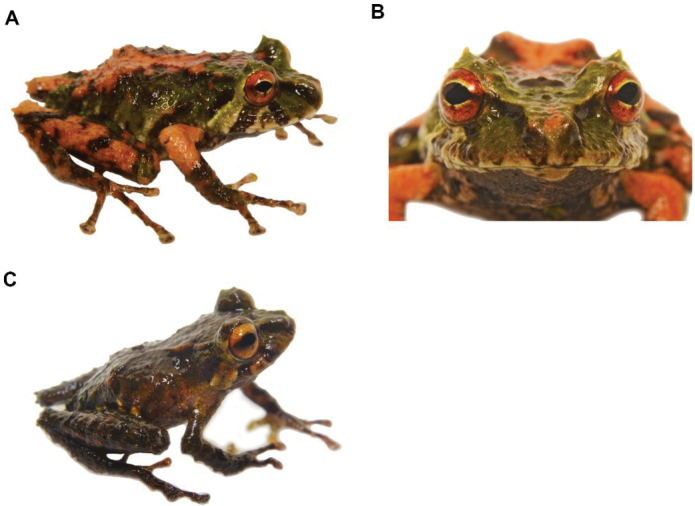
*Pristimantiseriphus* complex. Female (DHMECN 14437). **A** lateral view **B** frontal view, male (DHMECN 14436) **C** lateral view. Photographs by Mario H. Yánez-Muñoz.

#### ﻿ Pristimantis aff. gladiator

Taxon classificationAnimaliaAnuraStrabomantidae

9036D788-C87C-54ED-BCAC-BB091B771E92

Figure 22

##### Remarks.

Five female specimens (DHMECN 14446, 14447, 14448, 14484, 14705) with body sizes of 13.58 mm up to 19.03 mm, three male specimens (DHMECN 14449-14450, 1486) with body sizes of 12.6 mm up to 15.02 mm and one specimen with undetermined sex (DHMECN14485) with a body size of 11.35 mm, were collected on the forest floor between the leaf litter and on a bromeliad leaf 30 cm from the ground, in the montane forests of the Machay Reserve at elevations between 2220 m to 3000 m. This species has been recorded in other localities, such as Cerro Candelaria Reserve, Chamana Reserve and Tungurahua Volcano; however, some preliminary phylogenetic analyses suggest that northern and southern forms correspond to distinct near-related species (Franco, in preparation). According to references and historical records, the speci­mens assigned to *Pristimantisfestae* of the Tungurahua Volcano (Lynch and Duellman 1980) actually correspond to this new species, which, in its external morphology, is more similar to *Pristimantisgladiator*; however, the latter has a longer face-cloacal length and, according to osteological skull analysis, this species has other morphological differences.

**Figure 22.**
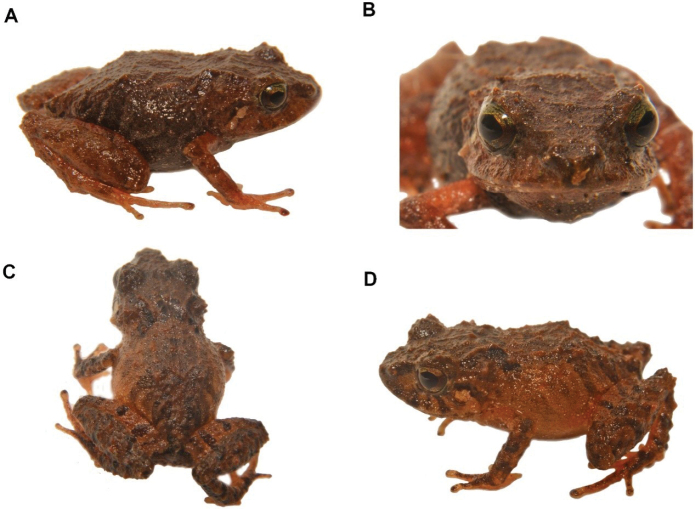
*Pristimantisaffgladiator*. **A** lateral view and **B** frontal view of females (DHMECN 14446) **C** dorsal view and **D** lateral view of male (DHMECN 14450). Photographs by Mario H. Yánez-Muñoz.

#### ﻿ Pristimantis aff. eriphus

Taxon classificationAnimaliaAnuraStrabomantidae

33422455-F303-5F87-B1DE-1B1EEAC325A8

Figure 23

##### Remarks.

Two male specimens (DHMECN 14443-14706) with a body size of 15.77 to 19.43 mm and one female specimen (DHMECN 14707) with a body size of 19.71 mm were collected on bush leaves at 100 cm from the ground, in the montane forests of the Machay Reserve at 2430 m elevation. The external morphology and colouration pattern are somewhat similar to *Pristimantiseriphus*; however, the latter has red eyes and white spots in the groin, as opposed to the yellowish spots and coppery yellow iris of the present species.

**Figure 23.**
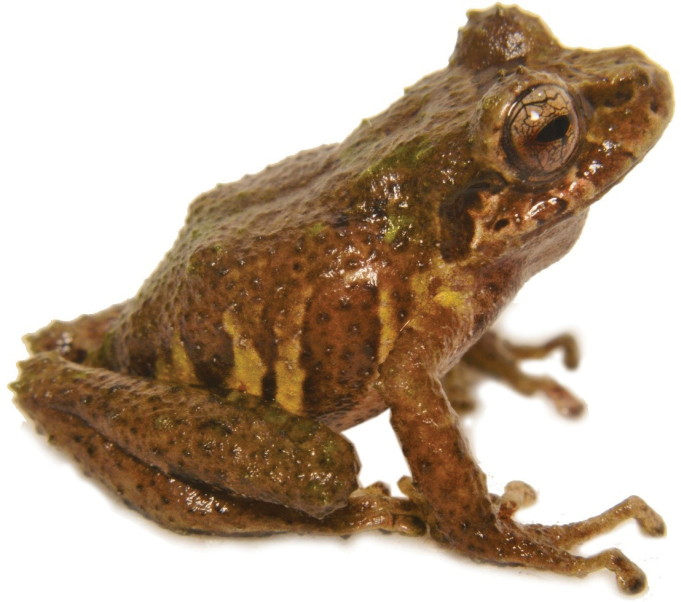
Prisitmantis sp. aff. eriphus. Lateral view of male (DHMECN 14443) Photograph by Mario H. Yánez-Muñoz.

#### ﻿ Pristimantis aff. bicantus

Taxon classificationAnimaliaAnuraStrabomantidae

933A847E-380D-54CF-A30E-3DF867E84C0B

Figure 24

##### Remarks.

A specimen (DHMECN 14444) of body size of 19.5 mm was collected on a dry branch 120 cm from the ground, in Machay Reserve montane forests at 2320 m elevation. This species is characterised by having small subconical tubercles on the upper eyelid and heel and brown dorsum.

**Figure 24.**
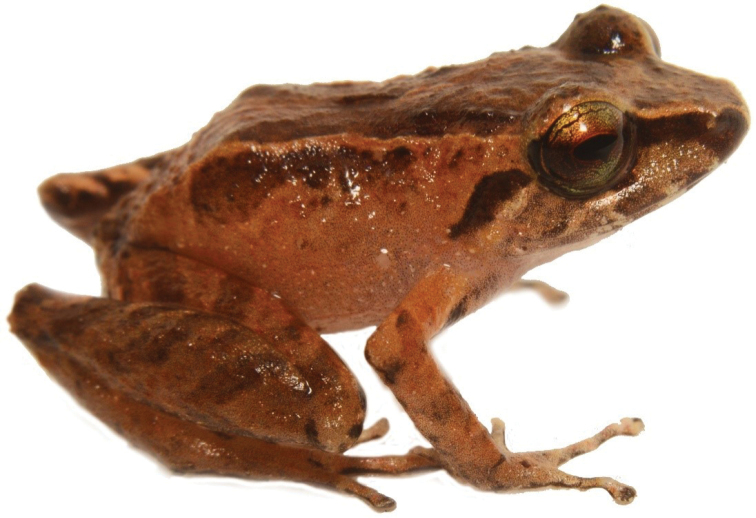
*Prisitmantis* sp. aff. bicantus. Lateral view (DHMECN 14444). Photograph by Mario H. Yánez-Muñoz.

#### ﻿ Pristimantis aff. tungurahua

Taxon classificationAnimaliaAnuraStrabomantidae

388EFEC2-66BB-5FC0-9014-AE84E0CB7D34

Figure 25

##### Remarks.

A specimen (DHMECN 14445) of body size of 21.04 mm was collected on a fern leaf 110 cm from the ground, in the montane forests of Machay Reserve at 2600 m elevation. This species is very similar to *Pristimantistungurahua* found in nearby localities, such as the Cerro Candelaria Reserve, Naturetrek-Vizcaya Reserve and Chamana Reserve. However, the Machay Reserve’s specimen differs in that it has patterns with white spots in the groin and ventral surfaces, as opposed to the red belly and dark colour of *P.tungurahua*.

**Figure 25.**
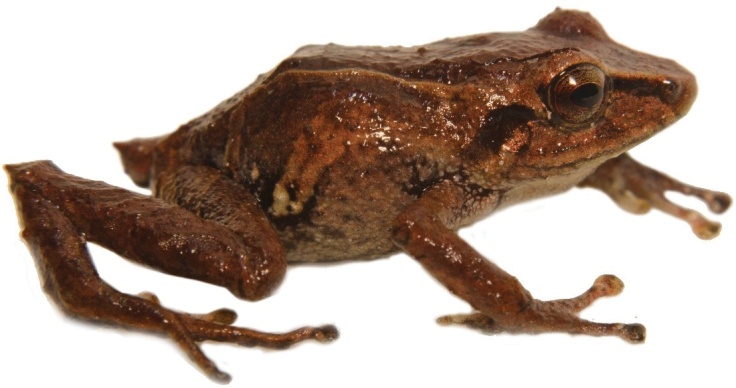
Pristimantis sp. aff. tungurahua (DHMECN 14445). Photograph by Mario H. Yánez-Muñoz

#### ﻿ Pristimantis

Taxon classificationAnimaliaAnuraStrabomantidae

sp. A

BE1325D7-6495-55DC-8465-0BB06AB2A89A

Figure 26

##### Remarks.

We recorded a specimen (DHMECN 14455) of body size of 11.67 mm, collected on bush leaves in the montane forests of the Machay Reserve at 3030 m elevation. This species is characterised by having conical tubercles on all dorsal surfaces with green and brown tones on the body and also presenting a rostral papilla at the tip of the snout.

**Figure 26.**
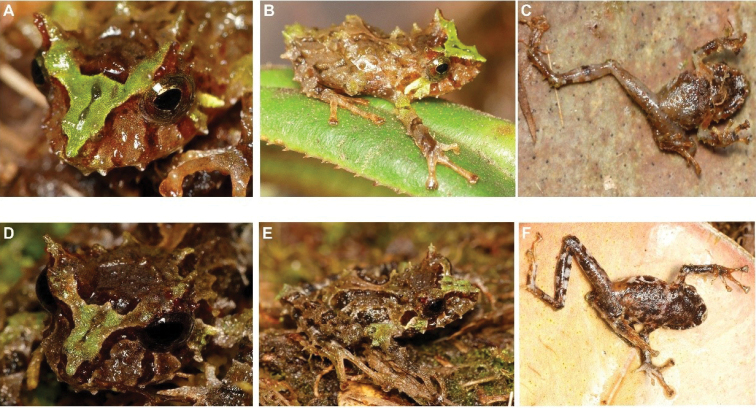
*Pristimantis* sp. A. (DHMECN 14455). **A** frontal view **B** lateral view **C** ventral view **D** detail of the head **E** lateral and dorsal view **F** ventral view of not collected specimen. Photographs by Lou Jost.

#### ﻿ Pristimantis

Taxon classificationAnimaliaAnuraStrabomantidae

sp. B

ADCC3111-6A52-5A40-A005-C9112A39E971

Figure 27

##### Remarks.

We recorded four specimens (DHMECN 14456-14457-14458-14459-14460-14461) with body sizes between 12.28 mm up to 16.85 mm, collected in the montane forests of the Machay Reserve at 3030 m elevation. This species is characterised by having subconical tubercles in the dorsal surfaces of the head and dorsum brown to copper with dark venter.

**Figure 27.**
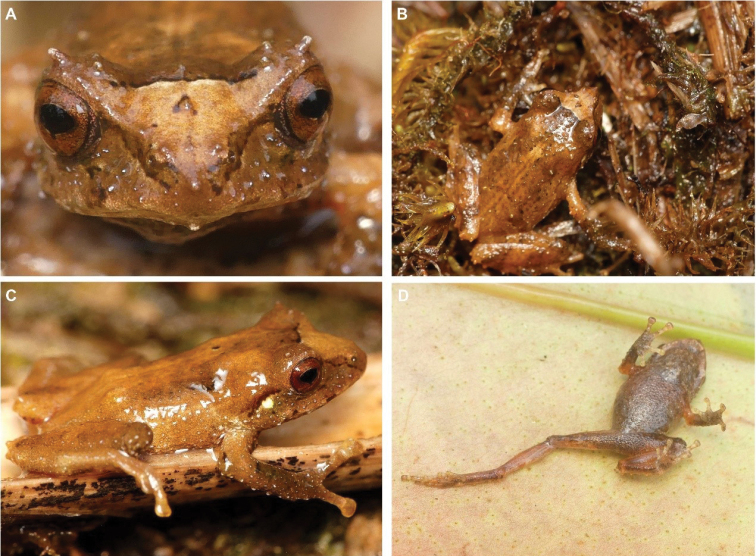
*Pristimantis* sp. B. **A** frontal view **B** dorsal view **C** lateral view (DHMECN 14457) **D** ventral view (DHMECN 14458). Photographs by Lou Jost.

#### ﻿ Pristimantis

Taxon classificationAnimaliaAnuraStrabomantidae

sp. C

06EF7A65-8EF2-5004-821C-F77C1A5D8405

Figure 28

##### Remarks.

We recorded four specimens (DHMECN 14462-14463-14464-14465) of body sizes between 15.10 mm and 22.93 mm collected in bromeliads in the montane forests of Machay Reserve. This species is characterised by having conical tubercles on the upper eyelid, dorsolateral folds and a distinctive colouration pattern on the flanks, groin and hidden surfaces of the thigh characterised by a brown background with white spots.

**Figure 28.**
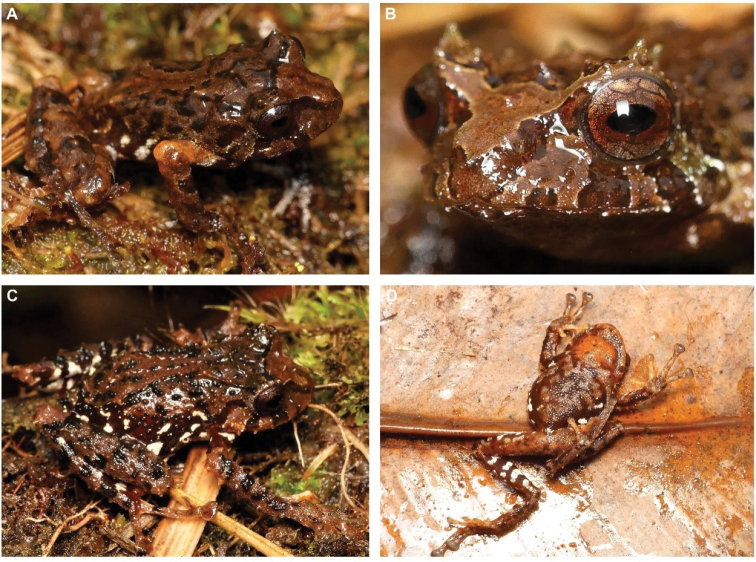
*Pristimantis* sp. C. (DHMECN14465). **A** lateral view and **B** frontal view **C** dorsal view (DHMECN 14463) and **D** ventral view (DHMECN 14462). Photographs by Lou Jost.

#### ﻿ Pristimantis

Taxon classificationAnimaliaAnuraStrabomantidae

sp. D

B449C9E8-F6A2-5E62-9934-D3E90D22FEA0

Figure 29

##### Remarks.

We recorded two female specimens (DHMECN 14466-14703) with body sizes of 34.94 mm to 39.44 mm, four juvenile specimens (DHMECN 14472-14473-14474-14475) with body sizes of 14.29 mm to 16.25 mm and six male specimens (DHMECN 14467-14469-14468-14470-14471, 14704) with body sizes of 20.35 mm to 27.5 mm. The specimens were collected on leaves and branches of shrubs, bromeliads, ferns, and bulrush between 50 cm and 175 cm from the ground, in the montane forests of the Machay Reserve at elevations between 1990 to 3000 m above sea level. This species in its external morphology resembles the *Pristimantisdevillei* group; however, *Pristimantis* sp. D exhibits tubercles on the upper eyelids and on the external edge of the tarsus.

**Figure 29.**
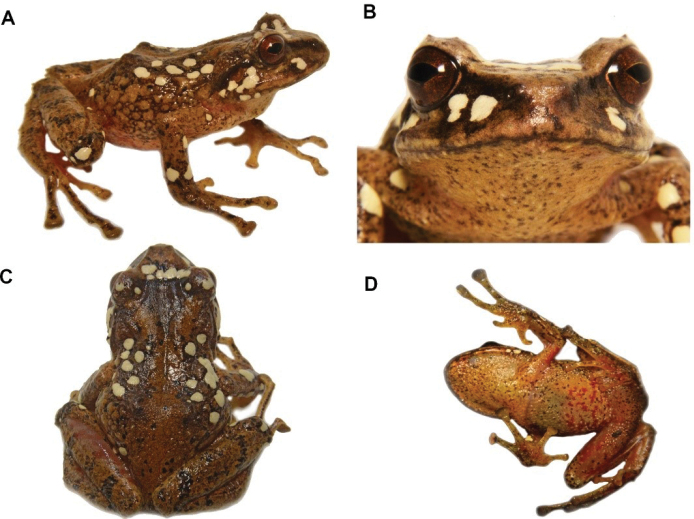
*Pristimantis* sp. D. Female (DHMECN 14466). **A** lateral view **B** frontal view **C** dorsal view **D** ventral view. Photographs by Mario H. Yánez-Muñoz.

#### ﻿ Pristimantis

Taxon classificationAnimaliaAnuraStrabomantidae

sp. E

21F02924-9150-5D6F-B072-DB66F2CE3C6E

Figure 30

##### Remarks.

A specimen (DHMECN 14476) with body size of 20.49 mm was collected on a bush leaf at 170 cm in the montane forests of MER at 2970 m elevation. Due to its external morphology, this species could be related to the *Pristimantislacrimosus* group; however, all known species of that group are found only below 2000 m elevation. This species could be the highest record for the *Pristimantislacrimosus* group.

**Figure 30.**
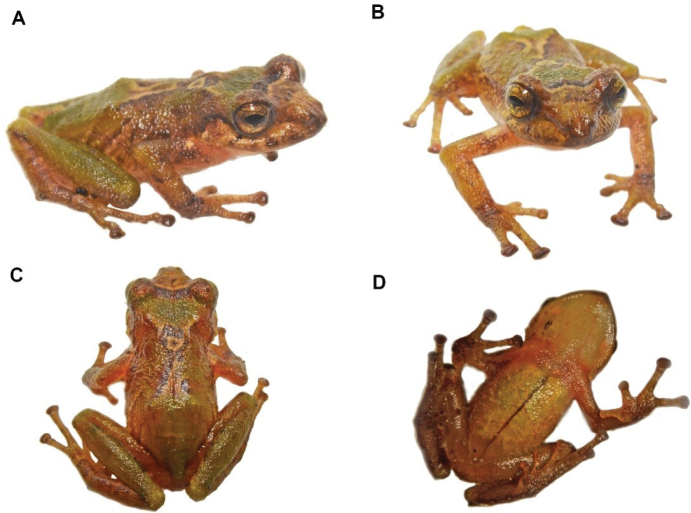
*Pristimantis* sp. E. (DHMECN14476). **A** lateral view **B** frontal view **C** dorsal view **D** ventral view. Photographs by Mario H. Yánez-Muñoz.

### ﻿New species

#### ﻿ Pristimantis maryanneae sp. nov

Taxon classificationAnimaliaAnuraStrabomantidae

F67F2ED7-9509-5765-AFAA-95EFB4430C41

http://zoobank.org/B1BBBC68-DE12-419B-BFDD-8DE2863EB1C5

**Figure 31.**
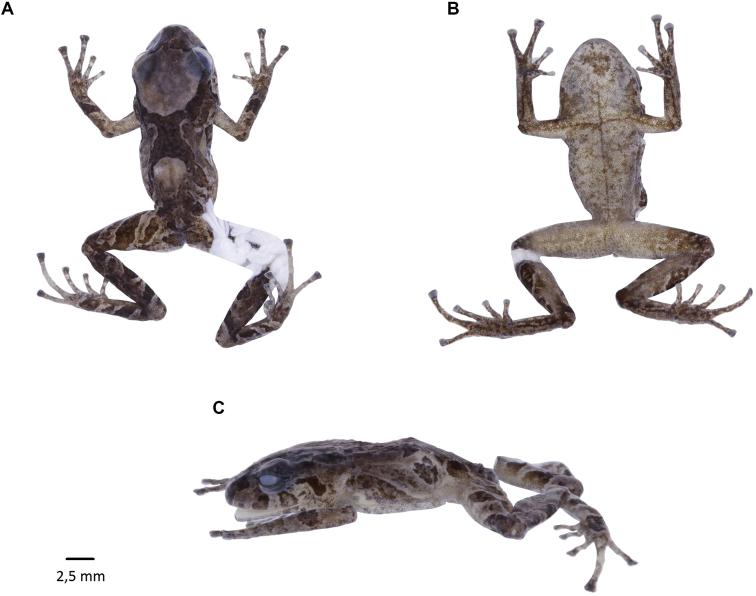
*Pristimantismaryanneae* sp. nov. (DHMECN 14454), adult, male holotype SVL = 17.9 mm **A** dorsal view **B** ventral view **C** lateral view. Photographs by Mario H. Yánez-Muñoz.

**Figure 32.**
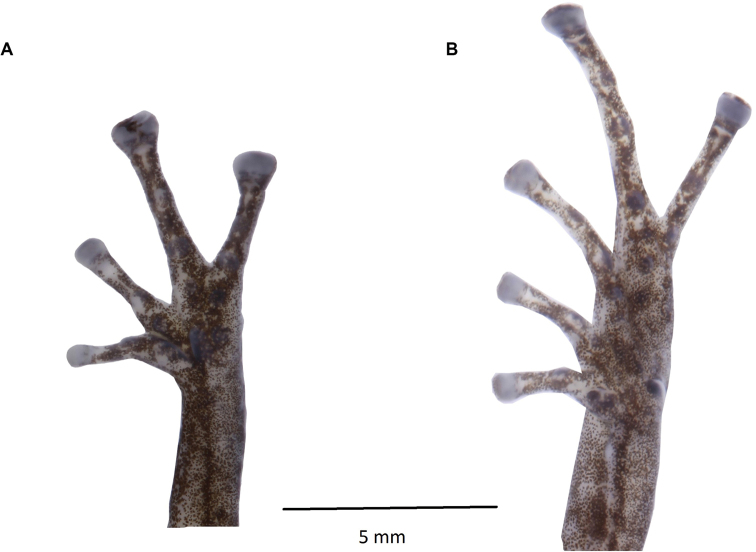
*Pristimantismaryanneae* sp. nov. (DHMECN 14454), adult male, holotype **A** palmar surface detail **B** plantar surface detail Photographs by Mario H. Yánez-Muñoz.

**Figure 33.**
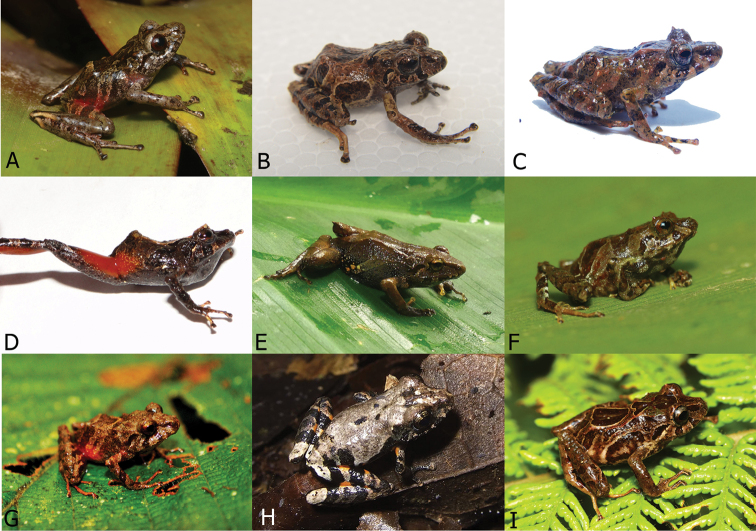
Live photograps of new species and comparison with similar *Pristimantis* frogs in the region **A***Pristimantisburtoniorum* sp. nov. (DHMECN 16220 from Machay Reserve Cerro Mayordomo.) **B***P.maryanneae* sp. nov. (DHMECN 14454 from Naturetrek Vizcaya Reserve) **C***P.prolatus* (DHMECN 16244 from El Encanto) **D***P.albujai* (DHMECN 12245 from Sardinayacu river) **E***P.tungurahua* (DHMECN 15224 from Naturetrek Vizcaya Reserve) **F***P.puruscafeum* (not collected from Cerro Candelaria Reserve) **G***P.sacharuna* (DHMECN 16723 from Rio Zuñag Reserve) **H***P.ventrimarmoratus* not collected from Río Zuñag Reserve **I***Pristimantis* sp (DHMECN 16250 from El Encanto) . Photographs Juan Pablo Reyes Puig, Mario Yánez Muñoz, Jorge Brito and Lou Jost.

Figures 31 , 32 , 33 Proposed standard English name: Maryanne’s Robber Frog Proposed standard Spanish name: Cutín de Maryanne

##### Material examined.

***Holotype*.**DHMECN 14454 (adult male Fig. 32), collected by Mario Yánez, Juan Pablo Reyes-Puig and Daniela Franco-Mena, in the Naturetrek Vizcaya Reserve, Ulba Parish, Baños township, Tungurahua Province, Republic of Ecuador (-1.357750, -78.393533; 2404 m elev.) on 26 February 2018.

***Paratypes*** (1 female, 3 males). DHMECN 14451 (♂ adult male), DHMECN 14453 (juvenile), with same location data as the holotype; DHMECN 14452 (♂ adult male) with same location data as the holotype, collected on 27 February 2018 and DHMECN 14550 (♀ adult female) with the same location data as the holotype, collected on 15 February 2018.

##### Generic placement.

We assign the new species to *Pristimantis*, based on having head about as wide as body; cranial crests absent; dentigerous process of vomers present; “S” condition of the adductor muscles; terminal discs on digits, bearing well-defined circumferential grooves, supported by T-shaped terminal phalanges; Toe V as long as, or longer than, Toe III; and subarticular tubercles not protruding (Hedges 2008).

##### Diagnosis.

*Pristimantismaryanneae* can be distinguished from other *Pristimantis* by the following character combination: (1) skin on dorsum finely shagreen, with flat and low warts, weak and fine sacral fold, composed of some low warts, two pairs of scapular tubercles diagonal aligned behind the eye; venter areolate with pustules, discoidal fold present; (2) tympanum absent, hidden beneath the skin; tympanic anulus visible under the skin measuring 25% of the eye diameter; (3) snout short, rounded in dorsal and lateral profile; (4) upper eyelid with 2–3 subconical tubercles (rounded in preservative); upper eyelid wider than interorbital distance; cranial crests absent; (5) dentigerous process of vomer present, oblique in outline with 2–3 oval teeth; (6) males without vocal slits, no nuptial pads; (7) Finger I shorter than II; digital pads expanded; (8) fingers with weakly defined laterals fringes; (9) forearms with small ulnar subconical tubercles; (10) heel bearing a small subconical tubercle; outer edge of tarsus bearing small subconical tubercles, inner tarsal fold absent; (11) two metatarsal tubercles, inner oval twice or three times larger than outer oval that is subconical; (12) toes without lateral fringes; supernumerary tubercles present, Toe V larger than III, does not reach distal subarticular tubercle of Toe IV; (13) dorsal colouration dark grey to grey with green marks, with transverse dark brown marks, with a chevron and irregular “H” shaped marks, flanks with cream diagonal bands, shanks with cream diagonal bands and light brown interspaces, hind-limbs with grey transverse bands and dark brown interspaces; ventral colouration dirty cream with a line along middle of the venter, chin and outer mandibula mottled with dark brown marks, iris light brown to grey with black reticulation and horizontal coppery stripe and (14) SVL males 17.61–17.8 mm; female 21.06mm.

##### Comparisons with other species.

(Fig. 33) The condition of the hidden tympanum is a distinctive characteristic that differentiates *Pristimantismaryanneae* from other *Pristimantis* within the eastern versant of the Andes in central Ecuador. Only *P.ventrimarmoratus* shares the condition of the tympanum; however, the ventral colouration of that species is composed of large black and white marks and the hidden surfaces have orange and yellow marks. *Pristimantis* sp from El Encanto have different iris and ventral colour patterns. *Pristimantismaryannaeae* does not exhibit flash colours on the venter, groin and hidden surfaces. Externally the new species resembles *Pristimantisverecundus* (Lynch and Burrowes 1990) and *P.mutabilis* (Guayasamin et al. 2015) from north-western Ecuador; however, these species have extremely opposite distributions, dorsolateral folds, reddish colours on the groin in *P.mutabilis* and both have a tympanum, in contrast to *P.maryaneae*.

##### Description of the holotype.

**Figure 34.**
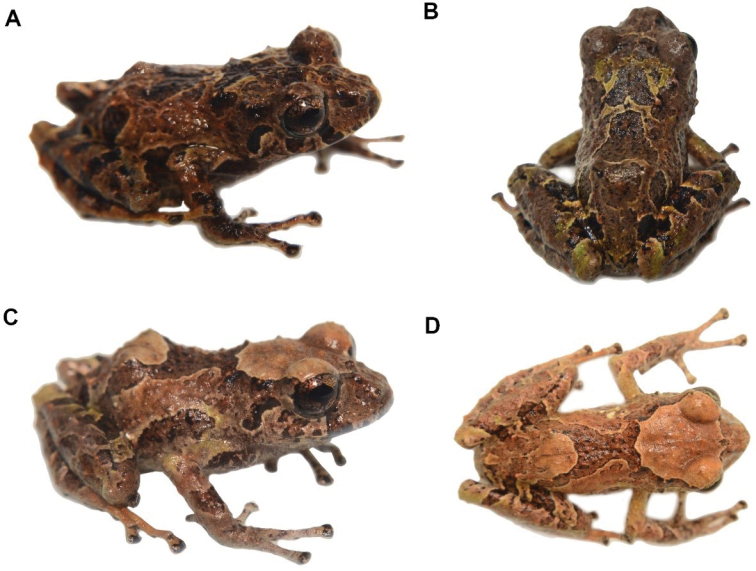
*Pristimantismaryanneae*, live photographs of Paratypes **A** lateral view and **B** dorsal view (DHMECN 14452) **C** lateral view and **D** dorsal view (DHMECN 14451). Photographs by Mario H. Yánez-Muñoz.

(Figs 31, 32) Adult male (measurements in mm): SVL 17.86; tibia length 9.07; foot length 9.18; head length 7.34; head width 6.78; upper eyelid width 1.97; interorbital distance 2.5; internarial distance 1.96; eye-nostril distance 1.81; eye diameter 2.35; tympanum diameter 0.79; hand length 5.08. Head longer than wide, length 41.6% SVL, wide 38% SVL; snout rounded in dorsal and lateral view (Fig. 31). Eye-nostril distance 10.13% SVL; canthus rostralis concave and loreal region slightly concave; nostril slightly protuberant orientated laterally; interorbital area flat, wider than the upper eyelid, upper eyelid measure 78.8% interorbital distance; cranial crests absent; occipital fold defined by the presence of two pairs of prominent subconical tubercles on the occipital and scapular regions; row of small rounded tubercles from the tip of the snout to the interorbital region; upper eyelid with three subconical tubercles (rounded in preservative); rounded tubercles scattered on the cantus rostralis and loreal region (Fig. 34); tympanic membrane undifferentiated from surrounding skin, tympanic annulus visible beneath the skin, rounded, laterally orientated, tympanum diameter 33.61% of the eye diameter, postrictal tubercles present, low; small choanae, rounded in outline, not covered by the palatal floor of maxilla; dentigerous process of vomer present oblique in outline; oval tongue longer than wide, 40% of it fixed to the mouth floor.

Skin on dorsum finely shagreen with rounded tubercles widespread; venter areolate with some pustules, discoidal fold present; anal ornamentation absent, with several rounded tubercles; forearms slender with two subconical ulnar tubercles and a row of subconical tubercles along the anterior edge (reduced or less evident in preservation effects); fingers with fine lateral fringes, palmar tubercle oval, the same size and shape as thenar; subarticular tubercles rounded and defined, with supernumerary tubercles at the base of each digit; digital pads truncated and expanded, twice as wide as the digit in fingers III, IV, on fingers I and II slightly wider than digit; all fingers have digital pads defined by circumferential grooves (Fig. 34).

Hind-limbs slender, tibia length 50.78% SVL, two subconical tubercles on the heel, a row of subconical tubercles on the outer edge of tarsus; inner tarsal fold present; toes with fine lateral fringes, without digital webbing, digital pads of the toes expanded, on toes IV and V twice as wide as digits and on toes I, II, III slightly more expanded than digit; low and rounded subarticular tubercles, supernumerary tubercles weakly defined and rounded; metatarsal tubercles present, inner oval three times the width of the outer tubercle that is rounded; toe V longer than III, not reaching to the base of distal subarticular tubercle of toe IV.

**Colour of holotype in life** (Fig. 34). Dorsal surfaces light brown to dark brown with dark marks, delineated by golden reticulations. Limbs banded with light brown and dark brown, delineated by golden tones. Throat light grey with small dark grey marks, an inverted triangle on the throat and chin, other ventral surfaces dark grey with light grey. Iris coppery/yellow with black reticulations.

**Colour of holotype in ethanol 70**% (Fig. 31). Head grey with an interorbital dark bar, nasal dark marks, a pair of subocular and supratympanic black bands. Dorsal pattern with irregular marks in several dark grey tones, delineated by irregular reticulations, a pair of dark marks forming ocellus on the posterior flanks. Anterior and posterior limbs banded from light grey to dark. Throat and venter with minute black points, ventral surfaces of forelimbs and hind-limbs grey.

**Variation** (Fig. 34). *Pristimantismaryanneae* shows dorsal variation in colour from light brown to dark brown, with some individuals bearing a head mask predominantly yellow; ventral surfaces can vary from small dense brown marks to grey tones. Variation in iris from coppery to grey coppery. Variation in morphometric measurements presented in Table 3.

**Table 3.** Measurements (in mm) of type series of *Pristimantismaryanneae* sp. nov. and *P.burtoniorum* sp. nov.

	Pristimantisburtoniorum sp. nov.		Pristimantismaryanneae sp. nov.	
Characters	Females (*n* = 3)	Males (*n* = 3)	Females (*n* = 1)	Males (*n* = 3)
**SVL**	20.8–27.3 (23.4 ± 2.7)	16.6–17.5 (17.1 ± 0.4)	21.1	17.6–17.9 (17.8 ± 0.1)
**TL**	11.7–12.9 (12.4 ± 0.5)	9.1–9.4 (9.3 ± 0.2)	10.3	8.7–9.1 0(8.9 ± 0.5)
**FL**	10.2–10.8 (10.5 ± 0.2)	7.8–8.8 (8.1 ± 0.4)	9.4	7.9–9.2 (8.4 ± 0.5)
**HW**	7.4–8.5 (8.1± 0.5)	6.2–6.5 (6.3 ± 0.2)	8.9	6.1–6.8 (6.4 ± 0.3)
**HL**	8.7–9.4 (9.1 ± 0.3)	6.7–7.7 (7.3 ± 0.4)	8.0	6.9–7.3 (7.1 ± 0.2)
**IOD**	2.7–2.8 (2.7 ± 0.01)	2.3–2.7 (2.4 ± 0.2)	2.4	2.2–2.5 (2.3 ± 0.2)
**EW**	1.6–2.4 (1.9 ± 0.3)	1.4–1.9 (1.7 ± 0.3)	1.5	1.5–2.0 (1.8 ± 0.2)
**IND**	2.2–2.2 (2.2 ± 0.0.1)	1.7–2.1 (1.8 ± 0.2)	2	1.7–2.0 (1.8 ± 0.1)
**EN**	2.3–2.5 (2.4 ± 0.1)	1.7–2.1 (1.8 ± 0.2)	2.1	1.5–1.8 (1.7 ± 0.2)
**TD**	1.1–1.2 (1.1 ± 0.06)	0.9–1.0 (0.9 ± 0.03)	1.0	0.7–0.8 (0.8 ± 0.02)
**ED**	3.0–3.1 (3.0 ± 0.01)	2.3–2.4 (2.3 ± 0.1)	2.6	2.2–2.4 (2.3 ± 0.1)

##### Distribution and natural history.

*Pristimantismaryanneae* is known only from the type locality, Naturetek Vizcaya Reserve, located at Ulba Parish, Baños township, Tungurahua Province, at 2400 m elevation in the eastern versant on the Andes in central Ecuador (Fig. 2), near the southwest limit of Llanganates National Park. This species was found in mature montane cloud forest (MAE 2012), characterised by a canopy of 25 to 30 m covered by epiphytes, orchids, bromeliads, bryophytes, and ferns. The bambusoid grass genus *Chusquea* was predominant in the area. The five known specimens of *Pristimantismaryanneae* were found in the lower stratum of the forest, sitting on leaves from 60 to 160 cm; one individual was found in leaf litter during the day, while all others were found on fern leaves at night.

##### Etymology.

Specific epithet is in recognition of Maryanne Mills (née Sawle), a zoologist from Perth, Australia. In 1986, she helped her husband, David Mills, set up the UK’s premier wildlife tour operator, Naturetrek and she has been based in England ever since. Her passion for the environment and its conservation has led Naturetrek to donate widely to this cause, including donations to World Land Trust which allowed EcoMinga Foundation to purchase more than 1,000 acres of Ecuadorian cloud forest, where this new species of terrestrial frog was discovered.

#### ﻿ Pristimantis burtoniorum sp. nov.

Taxon classificationAnimaliaAnuraStrabomantidae

838247D0-6AA1-54B6-AF9C-D72E998BF627

http://zoobank.org/0FAACD4F-D1D2-4913-A754-B01930417868

**Figure 35.**
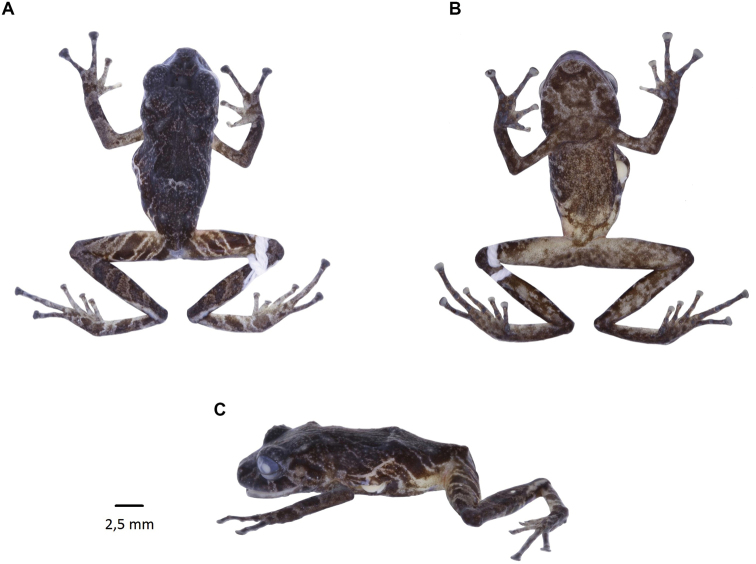
*Pristimantisburtoniorum* sp. nov. (DHMECN 14479), adult female, holotype, SVL = 22.6 mm **A** dorsal view **B** ventral view **C** lateral view. Photographs by Mario H. Yánez-Muñoz.

**Figure 36.**
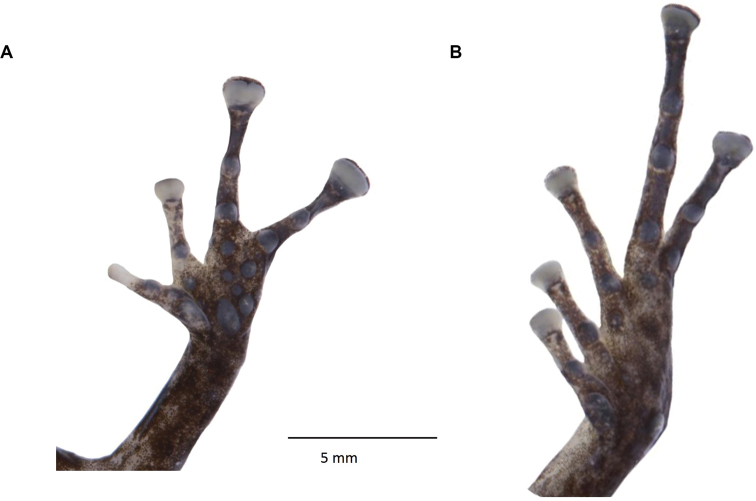
*Pristimantisburtoniorum* sp. nov. (DHMECN 14479), adult female, holotype **A** palmar surface detail **B** plantar surface detail. Photographs by Mario H. Yánez-Muñoz.

Figures 33 , 35 , 36 Proposed standard English name: Burtons’ Robber Frog Proposed standard Spanish name: Cutín de los Burton

##### Material examined.

***Holotype*.**DHMECN 14479 (adult Female, Fig. 35), collected by Mario Yánez-Muñoz, Juan Pablo Reyes-Puig and Daniela Franco-Mena, in the Machay Reserve, Rio Verde Parish, Baños township, Tungurahua Province, Republic of Ecuador (-1.370008, -78.268117; 2970 m elev.) on 2 March 2018.

***Paratypes*** (2 females, 3 males). DHMECN 14482 (♀), DHMECN 14447 (♀) y DHMECN 14480 (♂), DHMECN 14478 (♂), DHMECN 14481(♂), with same data as the holotype.

##### Generic placement.

We assign the new species to *Pristimantis*, based on having head about as wide as body; tympanic membrane differentiated t; cranial crests usually; dentigerous process of vomers usually present; “S” condition of the adductor muscles; terminal discs on digits, bearing well-defined circumferential grooves, supported by T-shaped terminal phalanges; comparative lengths of fingers I and II variable; toe V as long as, or longer than, toe III; and subarticular tubercles not protruding; (Hedges 2008).

##### Diagnosis.

*Pristimantisburtoniorum sp. nov*. is distinguished from all congeners by the following combination of characters: (1) skin on dorsum and flanks finely shagreen with a slightly defined mid-dorsal fold, which extends from the tip of the snout to the ventre; skin on ventre areolate, dorsolateral folds absent; discoidal fold present and defined; (2) tympanum present; tympanic membrane and annulus present, equivalent to 25% of the eye diameter; with a single subconic postrictal tubercle; (3) snout large and subacuminate in dorsal and lateral profile; with several small subconic tubercles along the upper mandibulae, more evident in females; (4) upper eyelid with 3–4 large subconic tubercles; one subconic interorbital tubercle, followed by a row of rounded tubercles along middle of the snout; upper eyelid wider than interorbital distance; cranial crests absent; (5) dentigerous process of vomer present, oval in outline with 4–5 teeth oval; (6) males lacking vocal slits, nuptial pads weakly defined; (7) finger I shorter than II ; expanded digital pads, extended in fingers II-IV; two times the width of the digits; (8) fingers with large lateral fringes; (9) forearms with small conic ulnar tubercles; (10) heel with a small conic tubercle; outer border of the tarsus with small conical tubercles, inner tarsal fold present, weakly defined in the first portion; (11) two metatarsal tubercles, inner oval twice size of the outer tubercle that is round-shaped; (12) toes with fine lateral fringes; plantar supernumerary tubercles present, toe V larger than III, not extending further than distal subarticular tubercle of toe IV; (13) dorsal colouration grey with transversal marks dark brown, legs and arms with diagonal bands dark brown with interspaces pink (red in life), flanks with oblique bands finely delineated by cream, hidden surfaces of the venter and groin red; ventral colour grey dense, marked by dark brown, throat and outer mandibulae with dark brown marks, brown-red iris and (14) SVL in males 16.61–17.45 mm; females 20.81–27.03 mm.

##### Comparisons with other species.

*Pristimantisburtoniorum* is characterised by the presence of red colouration in hidden surfaces of the hind-limbs, this colouration combined with banded patterns of brown and pink. The presence of tubercles on the upper eyelid, interorbital tubercle and a row of rounded tubercles along snout to the tip and a pale red venter with dark brown mottled pattern in life, easily distinguish the new species from other congeners occurring on the eastern Ecuadorian Andes, diagnostic morphological characters avoiding polymorphism of previously-known species (Fig. 33). Another rain frog with red colours on the ventral surfaces that may be confused with *P.burtoniorunm* is *P.tungurahua* (Reyes-Puig et al. 2010); however, this species has prominent calcars and dorsolateral folds; *P.sacharuna* (Reyes-Puig et al. 2015) exhibits red colours on the groin, but this is much darker red and is restricted to the groin and the digital pads are very much narrower than the new species. Other similar species in the upper Rio Pastaza watershed are *P.puruscafeum* (Reyes-Puig et al. 2014) and *P.prolatus* (Lynch and Duellman 1980); however, they have brown dorsal and ventral patterns with no flash colours on hidden surfaces and groins. Finally, *P.nigrogriseus* (Anderson 1945) has yellow hidden marks on the groin and *P.ventrimarmoratus* (Boulenger 1912) has black and white marks on the venter and the exhibits orange/yellow marks on the groin.

##### Description of the holotype.

(Figs 35, 36) Adult female. Measurements in mm: SVL 22.63; tibia length 12.85; foot length 10.56; head length 8.68; head width 7.36; upper eyelid width 1.93; interorbital distance 2.75; internarial distance 2.19; eye-nostril distance 2.32; eye diameter 4.7; tympanum diameter 2.5; hand length 7,17; head slightly wider than long (12.8 mm vs. 11.7 mm); head width 32.5% of SVL; head length 38% of SVL. Snout subacuminate in dorsal view, rounded in lateral profile (Fig. 35). Eye-nostril distance 10.25% of SVL; cantus rostralis straight, loreal region slightly concave; nostrils slightly protuberant, orientated laterally, interorbital area flat, wider than upper eyelid; upper eyelid length is 70% of the interorbital distance, cranial crests absent, occipital region prominent with a large rounded tubercle and two tubercles posterior and lower position. Upper eyelid with two rounded tubercles and other low. Row of small rounded tubercles along middle snout. Tympanic membrane differentiated from surrounding skin, tympanic annulus differentiated, low supratympanic fold with rounded shape, tympanum visible in dorsal view, laterally projected, tympanum diameter 35% of eye diameter, three subconical postrictal tubercles; choanae small, rounded in profile covered by the palatal shell of the maxilla; dentigerous processes of vomer present, oval on outline with 4–5 teeth; tongue longer than wide, oval shape, 60% of it fixed to the mouth floor.

Skin of the dorsum finely shagreen with scattered rounded tubercles; slightly defined dermal fold extending from the tip of the snout to the vent; venter areolate, discoidal fold present; without anal ornamentation and with low tubercles. Forearms slender with three lower ulnar tubercles and a row on the anterior region of the forearm, weakly defined or reduced by preservation effects, fingers with fine lateral fringes (Fig. 36), palmar tubercle oval three times larger than thenar tubercle that is rounded; subarticular tubercles of the fingers in hand rounded and elevated, supernumerary tubercles rounded at the base of each digit; digital pads truncated and expanded, twice width of fingers II,III, IV, in finger I slightly wider than the digit; all fingers have digital pads defined by circumferential grooves (Fig. 34).

Hind-limbs slender, tibia length 56.7% snout-vent length, small subconic tubercle on the heel with a row of three lower tubercles; inner tarsal fold present, outer edge of the tarsus with low tubercles; toes with fine lateral fringes, without digital webbing, toes digital pads expanded to twice the width of the digit; subarticular tubercles rounded, well defined and elevated, small supernumerary tubercles weakly defined; metatarsal tubercles present, inner oval double in size than outer that is rounded; toe V much longer than III, reaching base of distal subarticular tubercle of toe IV.

**Figure 37.**
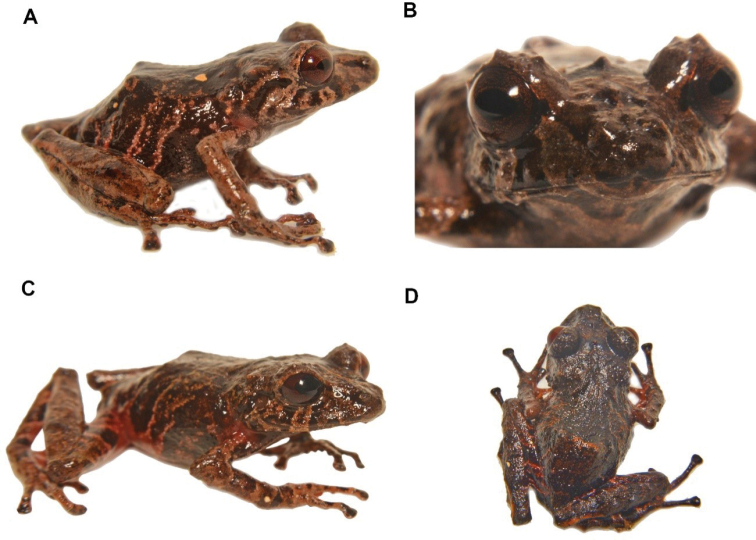
*Pristimantisburtoniorum***A** lateral view female paratype (DHMECN 14477) **B** frontal view, male paratype (DHMECN 14480) **C** groin view, female paratype (DHMECN 14482) **D** dorsal view of Holotype (DHMECN14479). Photographs by Mario H. Yánez-Muñoz.

**Colour of holotype in life** (Fig. 37). Dorsal surfaces dark brown, with irregular light brown marks, subocular and labial marks forming a banded pattern, body dark brown with light brown marks forming bands extending to the flanks, forelimbs and hind-limbs. Shanks and hidden surfaces of groin and armpit red, other ventral surfaces light brown with dark brown marks. Iris reddish-brown.

**Colour of holotype in ethanol 70**% (Fig. 35). Head and dorsum predominantly dark brown, banded with light brown and white lines extending to the flanks, forelimbs and hind-limbs as in the lips, a labial mask with a banded pattern is present. Groin and base of the venter and shanks red, other ventral surfaces mottled with light brown, chin with irregular dark brown and grey marks; forelimbs, hind-limbs, fingers and toes banded with brown and grey.

**Variation** (Fig. 37). Specimens of *Pristimantisburtoniorum* present a dorsal colouration with different tones of dark brown banded with light brown and cream, including variations with a thin mid dorsal line (Fig. 23). Variation in morphometric measurements are presented in Table 3.

##### Distribution and natural history.

*Pristimantisburtoniorum* is known only from the type locality in the Machay Reserve, Rio Verde Parish, Baños township, Tungurahua Province, Republic of Ecuador (Fig. 2) at an elevation of 2940 m. This locality is comprised of montane cloud forest (MAE 2012), with a canopy of 15 m with a dense layer of bryophytes and epiphytes and an understorey dominated by bromeliads of 30–60 cm in height from the forest floor. This is the predominant microhabitat for *Pristimantisburtoniorum*; all specimens were found hiding in the base of bromeliads leaves. Sympatric species are *Pristimantisfestae* complex, *P.buckleyi* complex, *Niceforonia* sp *and Hyloscirtus* sp.

##### Etymology.

Species epithet is the genitive plural of “Burton” in Latin, in recognition of John and Viv Burton, who founded and led the World Land Trust for most of its existence. Their impact on nature conservation is worldwide. Without the World Land Trust’s “Forests in the Sky” initiative, it would not have been possible for EcoMinga Foundation to establish the Machay Reserve and complete the Llanganates-Sangay Ecological Corridor.

## ﻿Discussion

The Upper Pastaza watershed shows a wide gradient of ecosystems, habitats and microhabitats for amphibian communities, with more diversity of species and reproductive strategies at eastern sites of study, mainly Bufonidae, Centrolenidae, Dendrobatidae, and Hylidae, highly influenced by Amazonian species groups and related to the availability of water resources for its reproductive modes, while western fauna appears to have more local endemism between localities north to south with dominant presence of Strabomantid frogs of the genus Pristimantis in montane and cloud forest localities; however, further research will complement our appreciation.

**Figure 38.**
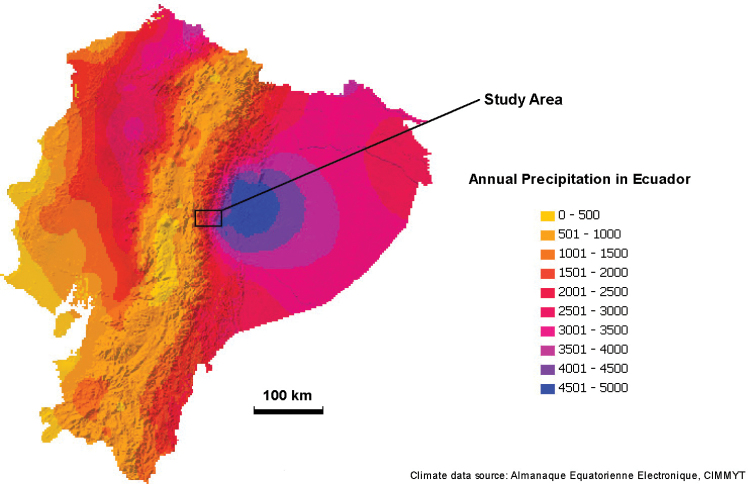
Precipitation in Ecuador. The indicated study area is approximately the area mapped in detail in Fig. 9.

Our study found high species richness in terrestrial frogs and filled some gaps in the distributions of some Andean arboreal frog lineages. We found a high proportion of candidate new species, of which two are described in this article. Previous studies have confirmed the high endemism and richness of the Upper Rio Pastaza watershed, specifically in the Llanganates-Sangay Ecological Corridor (Reyes-Puig et al. 2014; Reyes-Puig et al. 2015; Reyes-Puig et al. 2019a, b).

Our post-hoc analysis showed that SVL had a potentially important relationship with elevation, but the SVL values are widely scattered and the parameters of the best-fit least squares line for log_10_SVL as a function of elevation (in metres) have wide confidence intervals. One study investigating this same relationship, but within a population of a single species (in a very different habitat), showed a similar trend, with individuals decreasing in SVL by around 10% per thousand metres elevation change (Matthews and Miaud 2007). Another study, again with individuals of a single species, showed the opposite trend (Onn et al. 2018). Larger samples capturing a broader phylogenetic range of species will be needed to investigate this relationship further. It is important to mention that we made this exploratory analysis of our data after the original collection of information; therefore, we are not testing an explicit hypothesis of macro-evolutionary patterns of body size. We are describing a pattern reflected from the nature of our data; therefore and as we do not have a hypothesis to be tested, we did not perform an analysis that takes into account the error due to the phylogenetic correlation.

Despite our limitations, it is evident some examples of decreasing SVL with altitude in local communities of the study area, as an example, Bufonids genus *Rhinella* found in lower elevation sites, are larger and more slender than the Andean *Osornophryne* genus, with short SVL and limbs (Yánez-Muñoz et al. 2013). Another example could be addressed in the *Pristimantis* genus, with larger and more slender species in conspicillatus or lacrimosus groups in lower tropical zones, in comparison with smaller and short limbed species belonging to the Pristimantis myersi species group found in Andean ecosystems at high altitude (Lynch and Duellman 1997).

Our initial studies of the amphibian diversity pattern in the Upper Rio Pastaza watershed found a high altitudinal turnover of species, in bands with elevation amplitude less than 600 m. There was high horizontal heterogeneity as well. Over a distance of less than 40 km in the watershed, three communities share only 25% of the composition of their batracofauna (Yánez-Muñoz et al. 2013).

At first, it may seem surprising that peaks such as Vizcaya and Machay, separated by less than 15 km in a straight line and both on the same side of the Rio Pastaza, connected by contiguous forest at elevations subject to this study, are so dissimilar from each other and that, in spite of the enormous barrier of the deep and dry canyon of the Rio Pastaza, they have more similarity to communities at the same longitude south of the Pastaza. For example, based on our study, the Naturetrek Viscaya Reserve is more similar to the Chamana Reserve on the opposite side of the Rio Pastaza than it is to the nearby Machay Reserve on the same side of the Rio Pastaza (Fig. 9).

These results suggest that, while geographic barriers play a role in anuran distributions, an additional layer of complexity is added by small-scale spatial climate variations caused by the interaction of winds and topography; this distribution pattern has also been found in locally-endemic orchids, such as *Lepanthes* showing close relationship to the amount of precipitation (Jost 2004), in one of the more rainy areas in the Amazon Basin (Fig. 36).

While any specific microclimate may be patchy or isolated today, massive climate changes during the Pleistocene and Holocene almost certainly changed the topology of these patches, allowing the associated frogs to colonise new areas with the same microclimate, which later became isolated again as climate changed (Dodson 2003). This could be why some anuran species are found on both sides of the presently dry Rio Pastaza canyon in spite of their limited vagility (Hillman et al. 2014).

For fifteen years, we have collected and analysed information on amphibians in the Upper Rio Pastaza watershed and this has allowed us to establish species limits and describe several new lineages. However, we have refrained from issuing hypotheses on the evolutionary relationships of new species or making any deeper phylogenetic judgements. The present work will not be the exception. However, within our line of research, we can here announce a subsequent investigation that will summarise the phylogenetic, biogeographic and macro-ecological position of this diverse and poorly-known amphibian fauna of the tropical Andes.

## Supplementary Material

XML Treatment for Osornophryne simpsoni

XML Treatment for Centrolene buckleyi

XML Treatment for Gastrotheca

XML Treatment for Hyloscirtus sp. larynopygion

XML Treatment for Niceforonia

XML Treatment for Noblella naturetrekii

XML Treatment for Pristimantis bellae

XML Treatment for Pristimantis pastazensis

XML Treatment for Pristimantis tinguichaca

XML Treatment for Pristimantis tungurahua

XML Treatment for Pristimantis buckleyi

XML Treatment for Pristimantis eriphus

XML Treatment for Pristimantis aff. gladiator

XML Treatment for Pristimantis aff. eriphus

XML Treatment for Pristimantis aff. bicantus

XML Treatment for Pristimantis aff. tungurahua

XML Treatment for Pristimantis

XML Treatment for Pristimantis

XML Treatment for Pristimantis

XML Treatment for Pristimantis

XML Treatment for Pristimantis

XML Treatment for Pristimantis maryanneae

XML Treatment for Pristimantis burtoniorum

## ﻿Appendix I

List of amphibians recorded in Naturetrek-Vizcaya and Machay Reserves (Number of species is show in parentheses)

**Table:**

Taxon Amphibia (23)	Absolute abundance 97	Reserves: Machay Ecological Reserve (MR) and Naturetrek-Vizcaya Reserve (NVR)
BUFONIDAE (1)	–	–
**1. *Osornophrynesimpsoni***	**4**	**MR**
CENTROLENIDAE (1)	–	–
**2. *Centrolenebuckleyi* complex**	**1**	**NVR**
HEMIPHRACTIDAE (1)	–	–
**3. *Gastrotheca* sp.**	**2**	**NVR**
HYLIDAE (1)	–	–
**4. *Hyloscirtus* sp.**	**2**	**MR**
STRABOMANTIDAE (19)	–	–
**5. *Niceforonia* sp.**	**3**	**MR**
**6. *Noblellanaturetrekii***	**3**	**NVR**
**7. *Pristimantisbellae***	**4**	**MR**
**8. *Pristimantisbuckleyi* complex**	**2**	**MR**
**9. *Pristimantiseriphus* complex**	**8**	**MR**
**10. *Pristimantisburtoniorum* sp. nov.**	**7**	**MR**
**11. *Pristimantismaryanneae* sp. nov.**	**4**	**NVR**
**12. *Pristimantispastazensis***	**1**	**NVR**
**13 *Pristimantistinguichaca***	**1**	**MR**
**14. *Pristimantistungurahua***	**7**	**NVR**
**15. Pristimantisaff.eriphus**	**3**	**MR**
**16. Pristimantisaff.gladiator**	**9**	**MR**
**17. Pristimantisaff.bicantus**	**6**	**MR**
**18. Pristimantisaff.tungurahua**	**1**	**MR**
**19. *Pristimantis* sp. A**	**1**	**MR**
**20. *Pristimantis* sp. B**	**6**	**MR**
**21. *Pristimantis* sp. C**	**4**	**MR**
**22. *Pristimantis* sp. D**	**12**	**MR**
**23. Pristimantis sp. E**	**1**	**MR**

## ﻿Appendix II

List of Amphibians recorded in six localities of the Upper Pastaza Basin: Anzu Reserve (AR), Naturetrek-Cerro Candelaria Reserves (NCCR), Zuñac Reserve (ZR), Machay Reserve (MR), Naturetrek-Vizcaya Reserve (NVR), Chamana Reserve (CR).

**Table:**

TAXON AMPHIBIAN (92)	AR	ZR	CCR	MR	NVR	CR
AROMOBATIDAE (1)	–	–	–	–	–	–
1. *Allobateskingsburyi*	x	0	0	0	0	0
BUFONIDAE (5)	–	–	–	–	–	–
2. *Atelopuspalmatus*	0	x	0	0	0	0
3. *Rhinellafestae*	x	x	0	0	0	0
4. *Rhinellapoepigii*	x	0	x	0	0	0
5. *Rhinellamargaritifera*	x	x	x	0	0	0
6. *Osornophrynesimpsoni*	0	x	0	x	0	0
CENTROLENIDAE (4)	–	–	–	–	–	–
7. *Chimerellamariaelenae*	x	x	x	0	0	0
8. *Centrolenebuckleyi* complex	0	0	x	0	x	0
9. *Rulyranaflavopunctata*	x	x	0	0	0	0
10. *Nympharguscochranae*	x	0	x	0	0	0
DENDROBATIDAE (1)	–	–	–	–	–	–
11. *Ranitomeyavariabilis*	x	0	0	0	0	0
HEMIPHRACTIDAE (2)	–	–	–	–	–	–
12. *Gastrothecatestudinea*	0	0	x	0	0	x
13. *Gastrotheca* sp.	0	0	0	0	x	0
HYLIDAE (16)	–	–	–	–	–	–
14. *Dendropsophusbifurcus*	x	0	0	0	0	0
15. *Dendropsophusbockermanni*	x	0	0	0	0	0
16. *Dendropsophusparviceps*	x	x	0	x	0	0
17. *Dendropsophussarayacuensis*	0	0	x	0	0	0
18. *Dendropsophusminutos*	x	0	0	0	0	0
19. *Hyloscirtusphyllognatus*	x	x	0	0	0	0
20. *Hyloscirtus* sp.	0	0	0	x	0	0
21. *Boanaalmendarizae*	x	x	x	0	0	0
22. *Boanacinerascens*	x	0	0	0	0	0
23. *Boanageografica*	x	0	0	0	0	0
24. *Boanalanciformis*	x	0	0	0	0	0
25. *Osteceophalusfuscifacies*	x	0	0	0	0	0
26. *Osteocephalusverruciger*	x	x	x	0	0	0
27. *Osteocephalusmutabor*	x	0	0	0	0	0
28. *Scinaxruber*	x	0	x	0	0	0
29. *Trachycephaluscunauaru*	x	0	0	0	0	0
LEPTODACTYLIDAE (5)	–	–	–	–	–	–
30. *Adenomeraandreae*	x	0	0	0	0	0
31. *Engystomopspetersi*	x	0	0	0	0	0
32. *Leptodactylusandreae*	x	0	0	0	0	0
33. *Leptodactyluslineatus*	x	0	0	0	0	0
34. *Leptodactyluswagneri*	x	0	0	0	0	0
STRABOMANTIDAE (58)	–	–	–	–	–	–
35. *Niceforoniaelassodisca*	0	x	0	0	0	0
36. *Niceforonianigrovittata*	x	0	0	0	0	0
37. *Niceforonia* sp.	0	0	0	x	0	0
38. *Nobellalochites*	0	x	0	0	0	0
39. *Nobellanaturetrekii*	0	0	x	0	x	0
40. *Pristimantisaltamazonicus*	x	x	0	0	0	0
41. *Pristimantisaltamnis*	x	x	0	0	0	0
42. *Pristimantisardyae*	0	0	x	0	0	0
43. *Pristimantisbellae*	x	x	x	x	0	0
*44. Pristimantisbicantus*	0	x	x	0	0	0
45. *Pristimantisbuckleyi* complex	0	0	0	x	0	0
46. *10. Pristimantisburtoniorum* sp. nov.	0	0	0	x	0	0
47. *Pristimantischuruwiai*	0	0	x	0	0	0
48. *Pristimantiscroceoinguinis*	x	0	0	0	0	0
49. *Pristimantisdiadematus*	x	0	0	0	0	0
50. *Pristimantisenigmaticus*	0	x	0	0	0	0
51. *Pristimantiseriphus* complex	0	x	x	x	0	x
52. *Pristimantisganonotus*	0	x	x	0	0	0
53. *Pristimantisgaldi*	0	x	0	0	0	0
54. *Pristimantisincomptus*	x	x	0	0	0	0
55. *Pristimantislanthanites*	x	0	x	0	0	0
56. *Pristimantisloujosti*	0	0	x	0	0	0
57. *Pristimantismarcoreyesi*	0	0	x	0	0	x
58. *Pristimantismaryanneae* sp. nov.	0	0	0	0	1	0
59. *Pristimantismodipeplus*	0	0	0	0	0	0
60. *Pristimantisnigrogriseus*	0	0	0	0	0	0
61. *Pristimantispastazensis*	0	0	x	0	x	x
62. *Pristimantispetersioides*	x	0	x	0	0	0
63. *Pristimantispinchaque*	0	x	0	0	0	0
64. *Pristimantisprolatus*	x	x	0	0	0	0
65. *Pristimantispuruscafeum*	0	0	x	0	0	0
66. *Pristimantisquaquaversus*	x	x	x	0	0	0
67. *Pristimantisrubicundus*	0	x	0	0	0	0
68. *Pristimantissacharuna*	0	x	0	0	0	0
69. *Pristimantistinguichaca*	0	0	x	1	0	0
70. *Pristimantistungurahua*	0	0	x	0	x	x
71. *Pristimantisventrimarmoratus*	0	x	x	0	0	0
72. *Pristimantis w nigrum*	0	0	x	0	0	0
73. *Pristimantisyanezi*	0	x	0	0	0	0
74. Pristimantisaff.bicantus	0	0	0	x	0	0
75. Pristimantisaff.cajamarcensis	0	0	x	0	0	0
76. Pristimantisaff.cremnobates	0	x	x	0	0	0
77. Pristimantisaff.eriphus	0	x	0	x	0	0
78. Pristimantisaff.gladiator	0	0	x	x	0	x
79. Pristimantisaff.tungurahua	0	0	0	x	0	0
80. *Pristimantis* grp. *calcarulatus*	0	0	x	0	0	0
81. *Pristimantis* grp. *conspicilliatus*	x	x	x	0	0	0
82. *Pristimantis* grp. *devillei*	0	0	x	0	0	x
83. *Pristimantis* grp. *>orcesi*	0	0	x	0	0	0
84. *Pristimantis* sp. A	0	0	0	x	0	0
85. *Pristimantis* sp. B	0	0	0	x	0	0
86. *Pristimantis* sp. C	0	0	0	x	0	0
87. *Pristimantis* sp. D	0	0	0	x	0	0
88. *Pristimantis* sp. E	0	0	0	x	0	0
89. *Pristimantis* sp. F	0	x	0	0	0	0
90. *Pristimantis* sp. G	0	0	0	0	0	x
91. *Pristimantis* sp. H	0	0	0	0	0	x
92. *Strabomantiscornutus*	0	0	1	0	0	0
